# Supplementary material for: Emergent Properties of Tumor Microenvironment in a Real-Life Model of Multicell Tumor Spheroids
Source: PLoS One. 2010 Nov 30;5(11):e13942. doi: 10.1371/journal.pone.0013942 (PMC2994713; doi:10.1371/journal.pone.0013942)
Supplement: Text S1 — Including tables and additional references. (0.89 MB DOC) [file pone.0013942.s001.doc]

Supporting text for

Emergent Properties of Tumor Microenvironment in a Real-life Model of Multicell Tumor Spheroids

Edoardo Milotti^*^ and Roberto Chignola

*To whom correspondence should be addressed. E-mail: [milotti@ts.infn.it](mailto:milotti@ts.infn.it)

**This Supporting text includes:**

Materials and Methods

Tables S1 to S5

References

Introduction [3](#__RefHeading___Toc149365920)

A. Brief review of the single-cell model [3](#__RefHeading___Toc149365921)

The biochemistry of cells [4](#__RefHeading___Toc149365922)

Glucose uptake and retention by cells [4](#__RefHeading___Toc149365923)

Glucose-6-phosphate (G6P) utilization by cells [5](#__RefHeading___Toc149365924)

Glycogen storage [6](#__RefHeading___Toc149365925)

Glutamine (aminoacid) uptake [7](#__RefHeading___Toc149365926)

Oxygen uptake [8](#__RefHeading___Toc149365927)

ATP production and consumption [9](#__RefHeading___Toc149365928)

Lactate production [11](#__RefHeading___Toc149365929)

DNA synthesis [11](#__RefHeading___Toc149365930)

Protein synthesis [12](#__RefHeading___Toc149365931)

Mitochondrial proliferation [13](#__RefHeading___Toc149365932)

Dynamics of cell volume [13](#__RefHeading___Toc149365933)

Corrections due to internal and environmental pH [14](#__RefHeading___Toc149365934)

Other corrections [16](#__RefHeading___Toc149365935)

Cell phase transitions [17](#__RefHeading___Toc149365936)

B. Combined numerical solution of the diffusion and transport problem [21](#__RefHeading___Toc149365937)

C. Biomechanical modeling [23](#__RefHeading___Toc149365938)

Cell-cell forces [24](#__RefHeading___Toc149365939)

Brownian motion [26](#__RefHeading___Toc149365940)

Dynamical equations [28](#__RefHeading___Toc149365941)

Analysis of the simplified dynamical equations [28](#__RefHeading___Toc149365942)

Solution of the complete nonlinear system of dynamical equations [31](#__RefHeading___Toc149365943)

Mitosis [33](#__RefHeading___Toc149365944)

D. List of biophysical constants, parameter values [34](#__RefHeading___Toc149365945)

E. Some considerations on the computational complexity of the program [39](#__RefHeading___Toc149365946)

F. Snapshots from a simulation with the parameters of section E. [40](#__RefHeading___Toc149365947)

References [42](#__RefHeading___Toc149365948)

# Introduction

This supplementary text contains a systematic overview of the mathematical and computational details of the model introduced in the main text, and is divided in six parts. We start with a review of the single-cell model which summarizes the internal biochemical machinery included in the simulated cells: although this has already been described before [1,2], here we include some recent improvements and in the second part we summarize the setup of the numerical method for diffusion and transport processes. This is followed by a review of the dynamical interactions whereby cells interact mechanically with one another. Finally we list all the parameters used in the simulation program and include broad considerations on the computational complexity of the model. The last section contains a selection of images obtained in the simulations.

# A. Brief review of the single-cell model

The cells in the simulation program behave as automata that obey mostly deterministic rules, although they also include some circumscribed but important randomness. Each cell has many individual status variables that are listed in table S1, and steps through well-defined phases before dividing. After cell division the old cell is erased from the program’s cell list, the daughter cells are added to the list and each of them starts a new independent cell cycle. The cell cycle is sketched in figure S1.

We approximate cells as soft spheres of radius *r*, surrounded by a thin layer that represents the extracellular space. The extracellular space is essential for a proper description of diffusion of molecules like glucose that are transported across the cell membranes by facilitated diffusion, and the variables that characterize the extracellular space around each cell are also included in the list of status variables.

## The biochemistry of cells

The biochemical model of an isolated tumor cell developed in references [1] and [2] is outlined in figure S2 and comprises the following parts:

1. Glucose uptake and retention by cells
2. Glucose-6-phosphate utilization by cells
3. Glycogen storage
4. Glutamine (aminoacid) uptake
5. Oxygen uptake
6. ATP production and consumption
7. Lactate production
8. DNA synthesis
9. Protein synthesis
10. Mitochondrial proliferation
11. Dynamics of cell volume
12. Checkpoints and phase changes
13. Corrections due to internal and environmental pH
14. Other corrections

Now we describe in detail each item in the list.

### Glucose uptake and retention by cells

The equations that regulate the metabolism and transport of glucose are

(S.1a)

(S.1b)

where and denote respectively the mass of glucose inside the cell (C) and inside the surrounding extracellular volume (c).

The metabolic (*M_G_*) and the transport (*T_G_*) functions are respectively

(S.2a)

(S.2b)

where

*h, a2c, c2a* are correction factors that shall be specified later on, *S* is the cell surface, *V_C_* is the cell volume, *V_c_* is the extracellular volume, and finally the values ; ; *K_1_, K_2_, K_22_*, are cellular parameters (see below).

The metabolic function (*M_G_*) in equation (S.1a) corresponds to hexokinase and glucokinase activity^1^.

The first term on the rhs of equation (S.1b) describes diffusion on the disordered lattice of extracellular volumes; here *D_G_* is the glucose diffusion coefficient either in the extracellular space or in the enviroment (water) (see table S5), the *g_bc_*’s are numerical coefficients that belong to the discretization of the diffusion problem on the cell network (see section B), and the triangular bracket denotes the set of the adjacent extracellular spaces. This diffusion part is necessary for the consistency of the whole procedure: diffusion in the cell cluster and transport into and out of the cell are inescapably related (see section B and ref. 4).

### Glucose-6-phosphate (G6P) utilization by cells

The equation that regulates the G6P content of the cell is

(S.3)

where is the mass of G6P inside cell *C*, and the rates *g_i_* denote different paths of G6P utilization (see also figure S2 and reference [1])

(S.4a)

(S.4b)

(S.4c)

The underlying utilization model is straighforward, all the rates *g_i_* are simply proportional to G6P content of the cell, as this fits quite well the existing experimental data [1].

Moreover *t_pH_* and *SensO2* are correction terms (see below) and *coeffg1*, *coeffg2*, *coeffg3* are cellular parameters (see below).

The metabolic function (*M_G_*) has been defined above for glucose, and corresponds to glucose transformation into G6P.

### Glycogen storage

Glucose is partly stored as glycogen. Its production/consumption, follows the equation

(S.5)

where *Store* is the glycogen mass inside the cell, the rates *r_i_* correspond to different paths of *Store* utilization, the rate *p_11_* corresponds to glycogen production via the glutamine pathway (defined below), and finally the rate *g_3_* is associated to the glycogen production path from G6P conversion

(S.6a)

(S.6b)

(S.6c)

(S.6d)

Notice that these rates are modeled with simple Michaelis-Menten functions, where the substrate is the *Store* itself (for consumption) or glutamine (for production).

In addition to the symbols already defined in the previous sections, there are also other correction factors, *t_p11_*, *SensATP*, and *SensO2* (defined later). There is also a couple of additional parameters, *Kmd* and *Kmc*, and two molecular weights, *PM_G_* and *PM_ATP_* (all quantities identified by symbols like *PM_X_* are molecular weights): they are all listed in table S2. There are also two quantities related to ATP, *ATP_St_* and *ATPOx*, which are defined in the section on ATP.

### Glutamine (aminoacid) uptake

The equations that regulate the metabolism and transport of glutamine are

(S.7a)

(S.7c)

where and denote respectively the mass of glutamine inside the cell (C) and inside the surrounding extracellular volume (c). As for glucose, equation (S.7c) includes a diffusion term and *D_A_* is the diffusion constant of glutamine either in the extracellular space or in the enviroment (water) (see table S5).

The metabolic (*M_A_*) and the transport (*T_A_*) functions are respectively

(S.8a)

(S.8b)

where , and

(S.9)

is the rate of glutamine consumption for ATP production (see below), and the three double-substrate Michaelis-Menten rates correspond to protein production, DNA synthesis, and glutamine consumption for mitochondrial proliferation. In these formulas there are two additional correction factors, *a2cA* and *c2aA*, defined below, and several parameters, *VMAXA*, *VMAXP_A_*, *VMAXDNA_A_*, *VMAXM_A_*, *Kmp*, *KmDNA*, *KmM*, all listed below.

### Oxygen uptake

The equation for oxygen metabolism and diffusion is

(S.10)

where *D_O2_* is the oxygen diffusion coefficient either in water or in the cluster of cells (see table S5), and the metabolic function is

(S.11)

There is no extracellular quantity here, as oxygen diffuses normally over the cell and there is no specific transport process.

### ATP production and consumption

The differential equation for ATP is

(S.12)

where *ATPp* is the total ATP mass in the cell, and *ATPtot* is the total variation rate. *ATPtot* is a sum of several contributions:

- *ATPOx* (ATP production rate from oxidative phosphorylation)
- *ATPNOx* (ATP production rate from anaerobic glycolysis)
- *ATP2* (ATP production rate from glycogen (*Store*) )
- *ATP3* (ATP production rate from aminoacids (glutamine) )
- *ConsATP* (ATP consumption rate due to gluconeogenesis)
- *ConsATP_1_* (parameterization of the ATP consumption proportional to cellular volume)
- *ConsATP_2_* (ATP consumption rate for protein synthesis)
- *ConsATP_3_* (ATP consumption rate for DNA synthesis)
- *ConsATP_5_* (ATP consumption rate for mitochondrial proliferation)

which are defined by the equations

(S.13.a)

(S.13.b)

(S.13.c)

(S.13.d)

(S.13.e)

(S.13.f)

(S.13.g)

(S.13.h)

(S.13.i)

(S.13.j)

In these equations there are a few additional parameters, , , , , and , which are specified below.

Here we remark that some of these terms are representative of large classes of processes, in particular *ConsATP_1_* represents all those processes that utilize ATP and are extensively distributed over the cellular volume[[1]](#footnote-2).

### Lactate production

The equations that regulate the production and transport of lactate are

(S.14a)

(S.14.b)

where *D_AcL_* is the lactate diffusion coefficient either in the extracellular space or in the enviroment (water) (see table S5), and

(S.15a)

(S.15b)

where , and, as before, *a2cAcL* and *c2aAcL* are correction factors, while *VMAXAcL* and *KmAcL* are cellular parameters.

### DNA synthesis

The equation for DNA synthesis is

(S.16)

where *DNA* is the fraction of a complete DNA molecule, and is the rate of DNA synthesis from a double-substrate Michaelis-Menten term:

(S.17)

Here the glutamine mass (*m_A_*) represents the contribution of the other aminoacids as well.

### Protein synthesis

The equation for protein production is similar to (S.16), with the difference that now it represents a whole class of substances (all the proteins):

(S.18)

where is the total protein mass in the cell, and is the rate of protein production from a double-substrate Michaelis-Menten term:

(S.19)

The protein mass determines also the amounts (masses) of some specific proteins, pRb (retinoblastoma protein) and three cyclins (D, E, X = A+B):

pRb_fraction = 1.5e-2 times

cyclinD_fraction = 10.e-3 times

cyclinE_fraction = 3.e-3 times

cyclinX_fraction = 14.e-3 times

The amount of pRb that is carried over to daughter cells in mitosis is important because this indirectly determines the duration of the G1-phase, and we model the fluctuating duration of the G1-phase assuming that pRb is bound to parts of the nucleus that are partitioned binomially at mitosis. This modeling requires one additional cell parameter, *NUCLEAR_OBJ*, which is the (fictitious) number of parts to which pRb is bound.

### Mitochondrial proliferation

The number of mitochondria is described by the continuous variable *M*, which follows the differential equation

(S.20)

where is the rate of mitochondrial proliferation and is associated to a double-substrate Michaelis-Menten term which is proportional to the production of mitochondrial DNA:

(S.21)

where *VMAXM* is yet another cellular parameter.

Mitochondria are partitioned randomly between the daughter cells at mitosis. Experimental observations indicate that this partitioning follows a binomial distribution [5,6], however, in order to better reproduce the observed desynchronization of disperse cell populations [7], mitochodria are clustered in small groups – a noteworthy fact that is experimentally observed [8] – and it is these groups that are actually shared between cells; the clustering factor *ClusteringFactor* belongs to the list of cell parameters as well.

### Dynamics of cell volume

Cellular volume is a notoriously complicated cellular variable [9,10], and in particular it is related to osmotic pressure, and thus to the concentration of many substances inside cells. Here we take the total ATP mass as representative of this vast array of substances, and we assume that the cellular volume is partly determined by the total ATP mass. In addition, there are fixed volume contributions from the cell nucleus and from the organelles. The organelles themselves are variable in number, and again, we take the number of mitochondria as representative of the whole class of cellular organelles. These considerations yield the following – extremely simplified – formula for the volume of living cells:

(S.22)

where *DNA* is the fraction of synthetized DNA molecule as in eq. (S.16), while *C1*, *C2* and *V_min_* are cellular parameters. Since *V_min_* is the nuclear volume, eq. (S.22) takes into account the effective doubling of nuclear volume during the S-phase.

In the program we also assume that a cell can become apoptotic – for reasons explained below, in the section on cell-phase transitions – and after death the volume gradually shrinks [11] according to the equation

(S.23)

which has the exact solution

(S.24)

where *DVap* is yet another cellular parameter, and *V_0_* is the cell’s volume at death. This shrinking continues until and then stops: thus the cluster of cells eventually contains a sizeble volume of dead cells’ residues.

### Corrections due to internal and environmental pH

The equations above include several correction factors that modify the enzyme activity according to the internal and the extracellular pH

= pH inside cell C

= pH in the extracellular space

We plan to include in the program a rather complete model of cellular acidity, but at the moment the internal pH is fixed and the external pH is computed from a simple model of the buffering capacity of the standard nutrient solutions [2]. If [*AcL*] is the lactate concentration, then the pH of the enviroment and of the extracellular spaces is

(S.25)

where^2,12^ *BufCapEnv* = 0.19953 kg/m^3^.

The pH value is used to compute the following correction factors [2,13-16]:

= correction of metabolic rates (depends on internal pH, and thus at the moment it is fixed)

= fine tuning of *p*_11_ (depends on internal pH, and thus at the moment it is fixed)

= correction to transport of glucose from extracellullar space into cell

= correction to transport of glucose from cell to extracellullar space

= correction to transport of glutamine from extracellullar space into cell

= correction to transport of glutamine from cell to extracellullar space

= correction to transport of lactate from extracellullar space into cell

= correction to transport of lactate from cell to extracellullar space

The defining equations are:

(S.26a)

(S.26b)

(S.26c)

(S.26d)

(S.26e)

(S.26f)

(S.26g)

(S.26h)

These equations are smoothed versions of piecewise linear approximations of experimental data [2,13-16].

### Other corrections

There are a few additional correction factors related to oxygen and ATP [1,2]:

= correction to glucose transport due to oxygen concentration

= correction to glucose metabolism due to oxygen concentration

= this factor is used to switch ATP production between oxidative phosphorylation and anaerobic glycolysis

The equations that define these correction factors are [1,2]:

(S.27)

(S.28)

(S.29)

The equations for *h* and *SensATP* are smoothed versions of piecewise linear approximations, and *ATP_St_* is a standard rate (this is a cellular parameter as well), that acts as a threshold that separates the two regimes of oxidative phosphorylation and anaerobic glycolysis. There are a two more parameters in these definitions, *O2St*, the standard oxygen concentration, and *K_O2_*. *ATPOx* is the rate of ATP production from oxidative phosphorylation (defined above, in the section on ATP). The hyperbolic tangents that occur in the formulas do not have any special meaning, they are only practical approximations of smoothed step functions.

## Cell phase transitions

The transitions from one cell phase to the next are regulated by a complex biochemical machinery that we include only in small part, and as it interacts in deep ways with cell metabolism.

Each cell in the simulation is in a certain phase of the cell’s cycle, and there are specific rules that determine the transition from one phase to the next.

We define the following phases (sketched in fig. S1):

1. G1m phase: G1 phase prior to the commitment to enter S phase (variable duration);
2. G1p phase: G1 phase after the commitment to enter S phase (variable duration);
3. S phase (variable duration);
4. G2 phase (variable duration);
5. M phase (fixed duration);
6. Death (indefinite duration);

The dynamics of the G1m-G1p and of the G1p-S transitions is regulated by the pRb protein: pRb forms a complex with an enzyme E and carries 16 putative phosphorylation sites [17,18]. In this context, the enzymatic reaction catalyzed by E is a phenomenological model of the reactions controlled by the transcription factor E2F that associates with pRb in real cells.

The pRB-E complex is synthesized in the G2 phase and it is partitioned at random between the daughter cells at mitosis. At the beginning of the G1m phase the cyclin D protein (CycD) is expressed and it phosphorylates the pRB-E complex upon rapid association with specific cyclin-dependent kinases [17,18]. The phosphorylation event is assumed to occur following the reversible bimolecular interaction between cyclin D and the pRb complex: a detailed study has shown that the precise mechanism of pRb phosphorylation is irrelevant with respect to the dynamics of the system’s response [17]. Upon partial phosphorylation of the pRb protein, a fraction of E molecules are released and catalyze a reaction whereby a substrate S is converted into a product P. When the concentration of S falls below the threshold *Thresh_S_start* the expression of the cyclin E protein (CycE) is activated and this marks the progression of the cell from the G1m phase to the following G1p-phase. CycE participates to pRb phosphorylation upon instantaneous association with specific cyclin-dependent kinases and leads to the hyperphosphorylation of the pRb with full E detachment. When the concentration of S falls below the threshold *Thresh_S_stop* the cell progresses to the S-phase. At the end of the G1m- and G1p-phases, the cyclins CycD and CycE are destroyed by a proteolytic degradation that is assumed to occur faster than the considered reaction kinetics. The nature of pRb phosphorylation naturally introduces a threshold and a time delay in the reaction catalyzed by E with characteristic times comparable with the cell progression through the various cell cycle phases [19,20].

The program computes first the attachment probabability

(S.30)

and then the fraction and the number of molecules with at least *k* occupied phosphorylation sites:

(S.31)

(S.32)

where is the concentration of the pRb protein, and is the concentration of the phosphorylating complex, which – as explained above – we identify with the sum of the D and E cyclins (that we assume to be phosphorylated, in a cytoplasm with plenty of ATP). Here *N* and *k* are cell parameters (although in this case we do not expect them to change in different cell lines – see below).

Finally *pRb_ONOFFratio* is the ratio of the detachment-attachment rates (of the mediated phosphorylation process of pRb):

= *pRb_ONOFFratio*

We remark that all the quantities related to pRb phosphorylation process depend directly on pRb concentration, and thus on protein mass, and they do not require additional differential equations.

The concentration of the enzyme E (E2F) released by the configurational switch of the pRb protein is given by

(S.33)

and this determines the variation of the concentration of substrate S which leads to the threshold mechanism for the G1m-G1p commitment point

(S.34)

(where *k3MM* and *kmMM* are two additional cell parameters). The cell crosses the G1m-G1p commitment point when the concentration of the substrate S reaches a low value *Thresh_S_start*. A still lower value *Thresh_S_stop* corresponds to the G1-S checkpoint transition.

DNA synthesis takes place during the S-phase only, and the program contains a variable that takes care of synthesized DNA (measured in units of complete molecule). When this counter equals 1 the S-phase ends and the cell enters phase G2.

The G2-phase ends when there is a sufficient amount of cyclin A + cyclin B (at least *CycXThr*). Finally the M-phase has a fixed duration (*M_T_MEAN*).

In the program we also define a special cell phase which corresponds to death. We introduce an average death rate due to metabolites in the environment:

*death rate* = *a_R_* [*AcL*]*_C_* (S.35)

where [*AcL*]*_C_* is the lactate concentration inside the cell, so that the probability that a given cell survives during a timestep is

(S.36)

(this is the just the conventional expression for the survival probability in toxicology^21^).

A cell can also die if there are not enough nutrients: we check for this condition at the start of each phase, and if the ATP content of the cell is less than a minimum mass *ATPmin*, the cell dies. The value *ATPmin* is calculated assuming that any residual cytoplasmic volume – which in our minimal model of the cell’s volume depends on the total ATP content of the cell – must be at least as large as the total mitocondrial volume, then from the volume formula (S.22), we find

(S.37)

# B. Combined numerical solution of the diffusion and transport problem

In the previous section we have introduced several non-linear differential equations: these equations are used to simultaneously model both internal cell metabolism, and transport and diffusion. The equations have a rather conventional structure, as it often happens in biology, where the models are often concerned with enzyme action in the synthesis and destruction of molecular species, and in the regulation of transport of molecules into and out of the cell or cellular compartments.

The modeling of transport and diffusion is achieved by discretization of the diffusion problem: the cells’ centers provide a natural discretization scaffolding and we use a Delaunay triangulation to define proximity relations [22-28]. Figure S3 shows schematically the cells and their extracellular spaces, together with two simple examples of triangulation, both in 2D and in 3D. The molecular species in the program can be grouped in three different classes. The first class includes substances that are neither transported outside the cell nor diffuse to neighboring cells, and obey differential equations with the generic form

(S.38)

where is the mass of the substance in cell *C*, and is a function that depends on the current state of the cell (e.g., in the case of stored glycogen, this function is implicitly defined by equation (S.5) and is equal to ). In this case, the algorithmically stable solution derived from the implicit Euler method is

(S.39)

where is the mass of substance in the cell at the *n*-th timestep.

The second class of substances behaves like oxygen, which is metabolized by the cell, but is free to diffuse from cell to cell, and obeys equations with the generic form

(S.40)

where in addition to the metabolic term there is a diffusion term as well (and the sum runs over all adjacent cells *B* – here adjacency is defined by the Delaunay triangulation). Now the algorithmically stable solution derived from the implicit Euler method is equivalent to the Backward Differentiation Method method for partial differential equations [29], and writes

(S.41)

The last class of substances is like glucose, which is transported across the cell membrane by facilitated diffusion. This case requires two equations per cell

(S.42a)

(S.42b)

where the first equation describes internal metabolism and transport across the cell membrane into (or from) extracellular space, while the second equation describes the mass balance in the extracellular space, which exchanges mass with the cell and is linked to the rest of the system by diffusion (the extracellular space corresponding to cell *C* is denoted by a lowercase *c*, and the sum num runs over the adjacent extracellular spaces *b*). Now the algorithmically stable solution derived from the implicit Euler method produces the pair of equations

(S.43a)

(S.43b)

Finally, taking into account all substances, we are left with a large set of nonlinear equations like (S.39), (S.41), (S.43a) and (S.43b). These equations are solved iteratively with Newton-Raphson steps [30]; these steps usually converge, but may occasionally fail if the mass is very close to zero, and in this case they are substituted by steps performed with the secant method [30]. The convergence condition is that for all masses the inequality

(S.44)

holds, where is a given precision, i.e., the difference between two successive values in the iteration must be less than a given fraction of the estimated mass.

# C. Biomechanical modeling

Mechanical interactions between cells are – if possible – even more complex than the biochemical network described above, and we know that cells are viscoelastic structures which are actively shaped by the cytoskeleton [31].

The simulation work faces the following difficulties:

- existing theoretical models capture only part of the actual features of cells and are not really applicable to the complex reality of cells in clusters (e.g., reference [32] describes an interesting model of erythrocytes, which are however both simpler and different from other human cells; the model is also inapplicable here, because it would lead to an exceedingly high computational complexity);
- many measurements are inapplicable to the simulation of cells (pipette aspiration techniques [33-35], as well as optical and magnetic tweezers measurements [36,37] and experiments that utilize atomic force microscopes [38-45], provide interesting data, but in a contexts that are different from those that we want to simulate);
- cells adapt their shape both to environment and to internal events (the most notable of which is obviously the mitosis)
- cells live in a high-viscosity environment where both the internal viscosity of cells and the external viscosity of the extracellular environment matter.

Clearly, phenomenological approximations and parameterizations carry even more weight in this context. We approximate the mechanical behavior of cells as follows:

- cells are (soft) spheres, and are characterized by the position of their center (a 3D vector) and by their radius;
- we assume that cells are close-packed, and this leads to the introduction of an effective radius that is important in repulsive interactions with other cells;
- adhesion forces are usually very short-ranged, but here we assume a much larger range, and in this way we account for the cell’s extensibility and deformation;
- we assume that cells are surrounded by viscous material similar to that found in the extracellular matrix [46];

In the following paragraphs we explain all this in further detail.

## Cell-cell forces

We use a parameterization of the cell-cell forces that is somewhat similar to that used in references [47] and [48]. For small deformations of the cell membrane, it is reasonable to assume that it behaves as in the Hertz problem (interaction of two spherical membranes) or as in the Boussinesq problem (axisymmetric pressure on a flat membrane) [49,50]: in both cases the force is proportional to , where *x* is the relative deviation from the equilibrium position and is a constant related to the problem parameters. In the Hertz problem the intensity of the force is

(S.45)

where *R* denotes the radius, *d* is the distance of the two spheres’ centers, *E* is Young’s modulus, is Poisson’s ratio, and the subscripts denote sphere 1 or 2, so that in this case

(S.46)

and

(S.47)

Under compression the repulsive force (S.45) can be quite large, especially after mitosis: such large forces are not observed in real cells, and thus we assume that at small enough separations *d* the force flattens out and has a constant modulus. We set the position of this flattening so that we obtain the observed duration of mitosis.

When cells are drawn apart the force is attractive, because of adhesion molecules on the cell membrane [51]. The force range of the adhesion molecules is quite small (tens of nanometers) but here we assume a far larger range, as large as a few microns: in this way we account – albeit phenomenologically – for shape deformations in the case of attractive forces. Cell-cell adhesion depends on the number of links between adhesion molecules on both cell membranes [52], and is a probabilistic process: if we assume a roughly Gaussian probability density which depends on the relative deviation *x*, then

(S.48)

is the probability density that a link is detached at relative distance *x*, where and are parameters that must be adjusted. This means that the average number of detached links at relative distance *x* is proportional to the cumulative probability

(S.49)

Computing the error function in the simulation program leads to an increase of the computational load, and thus we replace the expression (S.49) with the approximate formula

(S.50)

(the hyperbolic tangent approximates the error function everywhere to better than 2% precision). Finally we take a force that is proportional both to the number of links and to the previously calculated shape :

(S.51)

The values of the parameters *E*, , , and are listed in table S4 and the modulus of the force is shown in figure S4; the direction of the force is always along the line that connects the cell centers.

## Brownian motion

Cells are small and Brownian motion, i.e. random molecular forces, could play an important role, however in the high viscosity medium of a cell spheroid we find that Brownian motion is negligible. It is easy to estimate this from the 1D Langevin equation for Brownian motion along *x*:

(S.52)

where *m* is the cell’s mass, is the friction coefficient, and is a white noise process which is associated to the molecular thermal motion. It can easily be shown that the mean square fluctuation [53] is

(S.53)

and does not depend on the particle mass. Now if we take cells in a high-viscosity environment, , and estimate the friction coefficient using the Stokes-Einstein formula for a cell with radius *r* ≈ 5 µm, we find , then at *T* ≈ 300 K the mean square fluctuation and the RMS fluctuation are

(S.54a)

(S.54b)

so that it takes a time *t* ≈ 10^4^ s for an RMS fluctuation of 1 µm to develop. It follows that the Brownian motion of cells is negligible in the case a simulation of tumor spheroids with reasonable time steps (the time step cannot in any case be longer than the duration of the shortest phase, which is the M-phase and lasts ~ 2000 s. moreover time steps should be shorter than ~ 100 s to provide a good sampling schedule of most biochemical processes).

## Dynamical equations

The dynamical equations for the motion of cells are similar to those of dissipative particle dynamics [54,55], without stochastic terms (since Brownian motion is negligible):

(S.55)

where the indices *n* and *k* denote cells, the sums are over all neighboring cells, denotes the mass, and are position and velocity vectors of the cell, is the environmental friction coefficient, is the friction between the *n*-th and the *k*-th cell, is an external force (like gravity), and finally is the force that cell *k* exerts on cell *n*.

### Analysis of the simplified dynamical equations

The equations (S.55) include a cell-cell friction that acts only along the line that joins the cells’ centers, and if we relax this constraint, the equations become somewhat simpler and easier to analyze:

(S.56)

If there are *N* cells, then the system of equations (S.56) is a linear system of 3*N* ordinary differential equations, and we can solve it using an implicit-explicit Euler method [56,57] (implicit over velocities, explicit over intercellular forces which are assumed to change slowly), so that the discretized version of the differential equation now writes

(S.57a)

(S.57b)

Equations (S.57a) are a linear system that could in principle be solved using one of the standard methods [29], however the computational complexity of the standard exact solutions is , and since *N* can be of the order of several hundreds of thousands or a few millions of cells, the computational burden of the exact solutions is huge. For this reason we resort to an approximate, iterative method (this holds for the solution of the complete differential system (S.55) as well). Formally solving the equations (S.57a) for , we find

(S.58)

and we can rewrite this as

(S.59)

where the index *i* identifies the *i*-th iteration. It is easy to see that the iterative algorithm converges: we start by subtracting two successive iterations

(S.60)

from which we obtain the series of inequalities

(S.61)

and convergence follows when we notice that

(S.62)

i.e., the maximum difference between successive iterations approaches zero for large *i*.

In the section on Brownian motion we have already taken a rather high environmental viscosity , which corresponds to the viscosity of the hyaluronate and collagen in extracellular spaces [58,59]; the viscosity of cell-cell interactions is even higher [31-45], of the order of . If we take cells with radius *r* ≈ 5 µm, so that *m* ≈ 5·10^-13^ kg, we note that and that , and we let = 100 s, then we find . Using this value we can estimate the worst-case convergence speed: from equation (S.62) we see that we achieve a ten-fold accuracy improvement after *s* steps, so that , i.e., . We remark here that this estimate depends critically on the environmental viscosity: we assume the viscosity of hyaluronate, but if we took a lower viscosity environment, like water with , we would find and , so that *s* ≈ 500000. In the case of a water environment, convergence of the iterative algorithm is so slow that it becomes totally impractical, but fortunately we can stick to the other, more favourable case.

### Solution of the complete nonlinear system of dynamical equations

The simplified equations (S.56) are useful to analyze algorithmic convergence, but the program utilizes the full equations (S.55). In this case the equations obtained from the implicit-explicit Euler method for the *x*-component of the velocity vector are

(S.63)

(similar equations hold for the *y* and *z* component of the velocity). These equations can also be put into matrix form

(S.64)

where

(S.65)

and

(S.66)

The formal solution of the equations (S.64) is

(S.67)

and this equation can be recast in the iterative form

(S.68)

where once again the index *i* identifies the *i*-th iteration.

## Mitosis

When a cell completes the M-phase it is replaced by two daughter cells. The program selects a random direction for the axis that joins the centers of the daughter cells, and then it computes the positions of their centers. Since the total volume of the daughter cells is equal to the volume of the mother, the radius of each daughter cell is roughly equal to 80% of the radius of the mother, i.e., the distance between the new centers is about (see figure S5). Here the centers are only representative of an “average” cell position and are used to compute forces; the new cells are actually compressed and deformed to fit in the original volume.

The distance also sets the maximum value of the repulsive force: indeed when the daughter cells are not surrounded by other cells, the distance of their centers must increase from about to in a time equal to the duration of the M-phase. For cells with radius this means that the distance traveled by each cell is about 3 *µ*m. If we take the total duration of the M-phase about 2000 s, then the average speed of each cell is about 1.5 nm/s, and the average repulsive force is 30 pN, taking a cell-cell viscosity as above.

# D. List of biophysical constants, parameter values

Table S1: list of cell status variables in the simulation program.

| ***basic informations*** | |
| --- | --- |
| name | cell name (identifier) |
| mark | optional label to select a subset of cells (i.e., cells with a marker of some kind) |
| type | cell phenotype (cell-specific list of parameters) |
| T | local cell temperature (not used now) |
| ***cell state*** |  |
| phase | cell phase |
| death_condition | label that records the reason of death (valid for dead cells only) |
| age | cell age (since birth) |
| phase_age | age of present cell phase |
| age_mother | age of mother at mitosis |
| n_mitosis | number of cell generations since start of simulation |
| ***geometric and topological informations*** | |
| x,y,z | cell position vector |
| vx, vy, vz | cell velocity vector |
| r | cell radius |
| surface | cell surface |
| volume | cell volume |
| mass | cell mass |
|  |  |
| volume_extra | volume of extracellular space surrounding cell |
|  |  |
| neigh | number of neighbors |
| vneigh | list of neighboring cells |
| vdist | distances to neighboring cells |
| vcsurf | contact surfaces with neighboring cells |
| gnk | vector of geometric factors |
| contact_surf | total contact area with neighboring cells |
|  |  |
| isonCH | boolean variable that is true if the cell is in contact with the environment |
| env_surf | cell-environment contact area |
| ***metabolic variables*** | |
| pHi | internal pH |
| M | number of mitochondria |
| G | mass of glucose inside cell |
| G6P | mass of G6P inside cell |
| O2 | mass of oxygen inside cell |
| store | mass of glycogen inside cell |
| A | mass of glutamine inside cell |
| AcL | mass of lactic acid inside cell |
| protein | total mass of proteins inside cell |
| prot_rate | total protein production rate |
| DNA | total amount of DNA produced by replication |
| DNA_rate | DNA replication rate |

*Table S1: list of cell status variables in the simulation program. (ctd.)*

| ***rates*** | |
| --- | --- |
| GAbsRate | glucose absorption rate |
| GConsRate | glucose consumption rate |
| AAbsRate | glutamine absorption rate |
| AConsRate | glutamine consumption rate |
| StoreFillRate | glycogen production rate |
| StoreConsRate | glycogen consumption rate |
| AcLRate | lactic acid production rate |
| AcLOutRate | lactic acid expulsion rate |
| ***ATP-related variables*** | |
| ATP_St | standard ATP |
| ATP_Ox | ATP production rate (by oxidative phosphorylation) |
| ATP_NOx | ATP production rate (by anaerobic glycolysis) |
| ATP2 | ATP production rate from the glycogen store |
| ATP3 | ATP production rate from glutamine |
| ConsATP | total ATP consumption rate due to metabolic activity |
| ConsATP_1 | ATP consumption rate associated to mitochondrial activity and other volume-dependent processes |
| ConsATP_2 | ATP consumption rate associated to the production of proteins |
| ConsATP_3 | ATP consumption rate associated to DNA production |
| ConsATP_4 | (reserved for future use) |
| ConsATP_5 | ATP consumption rate associated to mitochondrial proliferation |
| ATPtot | total ATP variation rate (this is the sum of all production and consumption rates) |
| ATPp | ATP pool (total mass of ATP inside cell) |
| ATPmin | minimum tolerable ATPp level (it depends on the size of cell: larger cells have a larger ATPmin level) |
| ATPstart | this variable stores the initial value of ATPp in the newly born cell |
| ATPprod | ATP produced in cell during the last simulation step |
| ATPcons | ATP consumed in cell during the last simulation step |
| ***variables that characterize the extracellular space around a cell*** | |
| pH | pH in extracellular space |
| G_extra | mass of glucose in extracellular space |
| A_extra | mass of glutamine in extracellular space |
| AcL_extra | mass of lactic acid in extracellular space |
| ***other variables*** | |
| SensO2 | fraction of oxygen available with respect to request |
| ConsO | oxygen consumption rate |
| ***proteins and DNA*** | |
| DNA_spread | individual variation of DNA production rate (this is a quite rough modeling of variable DNA synthesis because of individual DNA damages) |
| M_T | average duration of M-phase |
| pRb | total pRb mass inside cell |
| ConcS | molar concentration of substrate needed for thresholds |
| cyclinD | total cyclin D mass inside cell |
| cyclinE | total cyclin E mass inside cell |
| cyclinX | total mass of A and B cyclins inside cell |
| NpRbk | total number of active pRb molecules |

*Table S2* : list of the biophysical constants that are hard-coded in the program

| **Name of constant** | **Value** | **Description** |
| --- | --- | --- |
| N_AV_ | 6.022e23 | Avogadro constant |
| Faraday | 96485.34 Coulomb | Faraday constant |
|  |  |  |
| PM_G_ | 0.18 kg | Molecular weight of glucose |
| PM_ATP_ | 0.507 kg | Molecular weight of ATP |
| PM_O2_ | 0.032 kg | Molecular weight of O_2_ |
| PM_AcL_ | 0.090 kg | Molecular weight of lactate |
| PM_G6P_ | 0.270 kg | Molecular weight of G6P |
| PM_pRb_ | 110. kg | Molecular weight of pRb |
| PM_A_ | 0.146 kg | Molecular weight of glutamine |
| PM_prot_ | 66.476 kg | Average molecular weight of proteins |
| PM_cyclinD_ | 33.729 kg | Molecular weight of cycline D |
| PM_cyclinE_ | 47.077 kg | Molecular weight of cycline E |
| PM_cyclinX_ | 52. kg | Molecular weight of cycline A+B |
|  |  |  |
| Gibbs_ATP_ | 3.1·10^4^ J/mol | Gibbs free energy from ATP hydrolysis |
|  |  |  |

The following tables list the parameters that represent the the environment and the cell phenotype, and that the program reads from external files, and the diffusion and viscosity constants, that are still hard-coded in the simulation program:

*Table S3: Starting values for the environmental concentrations (standard atmospheric pressure and culture medium)*

| **Substance** | **Environmental**  **concentration** |
| --- | --- |
| O_2_ | 0.007 kg/m^3^ |
| glucose | 0.9 kg/m^3^ |
| glutamine | 0.4 kg/m^3^ |

*Table S4: Phenotype (cell parameters)*

| **Parameter name** | **Value** | **Notes and references** |
| --- | --- | --- |
| *VMAX1* | 2·10^-9^ kg·s^-1^·m^-2^ | [60]. |
| *VMAX2* | 1.2·10^-19^ kg·s^-1^ | [61]. |
| *VMAX22* | 1.2·10^-18^ kg·s^-1^ | [61]. |
| *VMAXA* | 1·10^-9^ kg·s^-1^·m^-2^ | [62]. |
| *VMAXP* | 3·10^-19^ kg·s^-1^ | Estimated in this work by considering that the average protein content of a cell is approx. 2.6·10^-14^ kg |
| *VMAXP_A_* | 1.018·10^-20^ kg·s^-1^ | Estimated in this work by considering that glutamine mass is approx. 5% of the total protein mass in the cell. See also [63]. |
| *VMAXP_ATP_* | 1.81·10^-18^ kg·s^-1^ | Estimated in this work by considering albumin (585 aminoacids, 584 aminoacid bonds) as the “average protein” and that 2 molecules of ATP are required for each bond. |
| *VMAXDNA* | 5·10^-5^ molecules/s | Estimated in this work taking 3·10^9^ base pairs/cell and assuming that on average the S-phase lasts 25000 s |
| *VMAXDNA_A_* | 5.847·10^-20^ kg·s^-1^ | Estimated in this work by considering that the entire duplication of the DNA requires 6·10^9^ glutamine molecules. See also [64]. |
| *VMAXDNA_ATP_* | 9.01·10^-20^ kg·s^-1^ | Estimated in this work by considering that, on average, the free energy of each phosphodiester bond in DNA is 5.3 kcal/mole. See, e.g., [65]. |
| *VMAXM* | 2·10^-3^ mitochondria/s | Estimated in this work, assuming an average number of 150 mitochondria per cell. See, e.g., [66,67]. |
| *VMAXM_A_* | 7.3·10^-23^ kg·s^-1^ | Estimated in this work by considering that, on average, there are 1000 molecules of mitochondrial DNA/cell and that each molecule is 16000 bp. See, e.g., [66]. |
| *VMAXM_ATP_* | 1.125·10^-22^ kg·s^-1^ | Estimated in this work as for *VMAXDNA_ATP_*. See above. |
| *K_1_* | 0.27024 kg·m^-3^ | [60]. |
| *K_2_* | 1.80 kg·m^-3^ | [61]. |
| *K_22_* | 1.80·10^-2^ kg·m^-3^ | [61]. |
| *KmA* | 0.023798 kg·m^-3^ | [68]. |
| *Ka* | 0.054 kg·m^-3^ | Derived from fitting the model to data in [69]. See also [1]. |
| *Kmc* | 0.096 kg·m^-3^ | Derived from fitting the model to data in [69]. See also [1]. |
| *Kmd* | 1.8·10^-2^ kg·m^-3^ | Derived from fitting the model to data in [69]. See also [1]. |
| *KmO2* | 7·10^-4^ kg·m^-3^ | Derived from fitting the model to data in [69]. See also [1]. |
| *Kmp* | 6.7·10^-3^ kg·m^-3^ | See ref. 2. In the present case, however, the value is expressed in concentration units. |
| *KmDNA* | 4.5·10^-4^ (kg·m^-3^)^2^ | See [2]. In the present case, however, the value is expressed in concentration units. |
| *KmM* | 1.46·10^-2^ (kg·m^-3^)^2^ | Estimated in this work as for *VMAXM_A_* (see above). This value is calculated at equilibrium for standard concentrations. |
| *coeffg1* | 7.5·10^-3^ s^-1^ | Derived from fitting the model to data in [69]. See also [1]. |
| *coeffg2* | 1.08·10^-3^ s^-1^ | Derived from fitting the model to data in [69]. See also [1]. |
| *coeffg3* | 6.7·10^-4^ s-1 | Derived from fitting the model to data in [69]. See also [1]. |
| *coeffr1* | 3·10^-20^ kg·s-1 | Derived from fitting the model to data in [69]. See also [1]. |
| *ATP_St_* | 2.3·10^-18^ kg·s-1 | Derived from fitting the model to data in [69]. See also [1]. |
| *V_min_* | 0.9·10^-16^ m^3^ | Derived from data in [70] considering that the cell nucleus occupies between 4% and 14% of cell volume. See also [1]. |
| *DVap* | 3·10^-6^ s^-1^ | Estimated in this work. See also [11]. |
| *VMAXAcL* | 9.58·10^-8^ kg·s^-1^·m^-2^ | [71-73]. |
| *KmAcL* | 0.40536 kg·m^-3^ | [71-73]. |
| *M_T_MEAN* | 1800. s | [1]. |
| *v_work_* | 1.5·10^-2^ kg· s^-1^·m^-3^ | Estimated in this work by fitting of tumor cell growth data |

*Table S4: Phenotype (cell parameters). (ctd.)*

| Parameter name | Value | Notes and references |
| --- | --- | --- |
| *k_pRb* | 10 | [17]. |
| *N_pRb* | 16 | [17]. |
| *pRb_ONOFFratio* | 1·10^-6^ | [74]. |
| *pRb_fraction* | 1.5·10^-2^ | This paper by data fitting. See also [2]. |
| *cyclinD_fraction* | 1·10^-2^ | This paper by data fitting. See also [2]. |
| *cyclinE_fraction* | 3·10^-3^ | This paper by data fitting. See also [2]. |
| *cyclinX_fraction* | 1.4·10^-2^ | This paper by data fitting. See also [2]. |
| *ConcS_0* | 1·10^-3^ mol/l | [19,20]. |
| *Thresh_S_start* | 0.8 | [2]. |
| *Thresh_S_stop* | 0.05 | [2]. |
| *k3MM* | 1·10^4^ s^-1^ | [19,20]. |
| *KmMM* | 1·10^-3^ mol/l | [19,20]. |
| *NUCLEAR_OBJ* | 46 | Estimated in this work by fitting of tumor cell growth data |
| *ClusteringFactor* | 15 | Estimated in this work by fitting of tumor cell growth data |
| *CycXThr* | 0.8·10^-16^ kg | [2]. |
| *C1* | 0.074 m^3^ kg^-1^ | Inverse of standard ATP concentration (see above) |
| *C2* | 2·10^-19^ m^-3^ | Average volume of mitochondria. See [75]. |
| *a_R_* | 1.2·10^-5^ m^3^·kg^-1^·s^-1^ | Estimated in this work by fitting of tumor cell growth data |
| *YoungMod* | 1000. Pa | Young’s modulus. The elastic properties of cells are quite variable: according to [38], Young’s modulus of living cells spans a very large range (1-100 kPa). Here we choose a value which is at lowest extreme of this range, and agrees with measurements in ref. [41,42,44,45]. |
| *PoissonRatio* | 0.5 | Poisson’s ratio. This number relates axial and transverse strain and lies in the range (-1,0.5) [76]. Here we choose the highest possible value, 0.5, which corresponds to incompressible cells (as in [44]). |
| *density* | 1070. kg·m^-3^ | [77,78]. |
| *viscosity* | 200. Pa·s | Cytoplasmatic viscosity, see [34,79]. |
| *adhesion_range* | -0.5·(cell radius) | This work, by fitting of tumor cell growth data. |
| *adhesion_decay* | 2. | This work, by fitting of tumor cell growth data. |
| *packing_factor* | 0.9047 | Adimensional correction that takes into account the interstitial volume (linear packing factor for an arrangement of spheres [80,81]) |
| *extension_coeff* | 1.1 | Correction factor that makes cells a little softer (shall be removed in a future version of the program with a better definition of the Poisson’s ratio). This work by fitting of tumor cell growth data. |
| *extvolume_thickness* | 1.0·10^-7^ m | Thickness of extracellular space surrounding each cell. Direct measurements from micrographs of tumor spheroids. |
| *extvolume_fraction* | 0.3 | This is the fraction of external volume that is actually accessible to diffusing molecules [82-84]. |
| *tph_slope* | 2.73 | This work, by fitting of tumor cell growth data. See also [2, 13-16]. |
| *tph_thr* | 6.55 | This work, by fitting of tumor cell growth data. See also [2, 13-16]. |
| *tp11_slope* | 10.91 | This work, by fitting of tumor cell growth data. See also [2, 13-16]. |
| *tp11_thr* | 6.9625 | This work, by fitting of tumor cell growth data. See also [2, 13-16]. |
| *a2c_slope* | 2.42 | This work, by fitting of tumor cell growth data. See also [2, 13-16]. |
| *a2c_thr* | 6.92 | This work, by fitting of tumor cell growth data. See also [2, 13-16]. |
| *c2a_slope* | 2.42 | This work, by fitting of tumor cell growth data. See also [2, 13-16]. |
| *c2a_thr* | 6.92 | This work, by fitting of tumor cell growth data. See also [2, 13-16]. |
| *a2cA_slope* | 2.42 | This work, by fitting of tumor cell growth data. See also [2, 13-16]. |
| *a2cA_thr* | 6.92 | This work, by fitting of tumor cell growth data. See also [2, 13-16]. |
| *c2aA_slope* | 2.42 | This work, by fitting of tumor cell growth data. See also [2, 13-16]. |
| *c2aA_thr* | 6.92 | This work, by fitting of tumor cell growth data. See also [2, 13-16]. |
| *]a2cAcL_slope* | 1.5 | This work, by fitting of tumor cell growth data. See also [2, 13-16]. |
| *a2cAcL_thr* | 7. | This work, by fitting of tumor cell growth data. See also [2, 13-16]. |
| *c2aAcL_slope* | 1.5 | This work, by fitting of tumor cell growth data. See also [2, 13-16]. |
| *c2aAcL_thr* | 7. | This work, by fitting of tumor cell growth data. See also [2, 13-16]. |

*Table S5: Other parameters that at present are hard-coded in the program.*

| **Name** | **Value** | **Notes and references** |
| --- | --- | --- |
| Diff_W_G | 7·10^-10^ m^2^/s | glucose in water [82]. |
| Diff_ES_G | 2·10^-10^ m^2^/s | glucose in the extracellular spaces [82,85]. |
| Diff_W_A | 3·10^-10^ m^2^/s | glutamine in water [82]. |
| Diff_ES_A | 7·10^-11^ m^2^/s | glutamine in the extracellular spaces [86]. |
| Diff_W_AcL | 3·10^-10^ m^2^/s | lactate in water [82]. |
| Diff_ES_AcL | 1.4·10^-11^ m^2^/s | lactate in the extracellular spaces [87]. |
| Diff_W_O2 | 3.2·10^-9^ m^2^/s | oxygen in water [48]. |
| Diff_ES_O2 | 7·10^-11^ m^2^/s | oxygen in the extracellular spaces. This work, by fitting of tumor cell growth data. |
| VISCOSITY_ENV | 1. Pa·s | viscosity of the extracellular space [58]. |

# E. Some considerations on the computational complexity of the program

This kind of simulation is a real computational challenge. Although the present version of the program is not completely optimized, it has been designed with computational complexity in mind. On average, the time complexity of each individual time step is , where *N* is the number of cells, and this is determined by the Delaunay triangulation [88-91].

However the number of cells grows exponentially, at least in the initial phase of spheroid growth, and thus the computational complexity of each simulation step is proportional to , where *t* is the simulated time and *T* is the average duplication time of cells. The CPU time required to simulate the time span is modeled by the expression

(S.69)

where and are experimental parameters that we determine from actual simulation runs, and where we introduce a slightly superlinear term – suggested by the maximum complexity of Delaunay triangulations in the plane – to account for a superlinear growth term with respect to *N*. Mature spheroids slow down their growth rate and we find that at this later stage is roughly proportional to the number of cells:

(S.70)

where and are yet other parameters that we determine from actual runs. This also means that on the whole the total computing time is roughly proportional to .

The actual values of , ,, and depend on the simulation parameters: figure S6 shows the CPU time (s) it takes for one hour of simulated time vs. *N*, in a run where the precision of the global solution of the problem of diffusion, transport and metabolism is fixed at 1%, and the simulation time step is  = 50 s. This run has been carried out on a MacPro computer (with an Intel Xeon 3500/W3520 quad processor at 2.66 GHz – and where a single core has been used, the program is not yet multithreaded). Figure S7 shows the total CPU time vs. *N*: fitting this curve we can estimate the total CPU time needed to simulate larger spheroids.

# F. Snapshots from a simulation with the parameters of section E.

The final supplementary figures are higher-resolution pictures of central slices from a simulation with the parameters listed in the previous section. The figures correspond to different simulated times.

- figures S8-10: oxygen concentration (color mapping, blue=low concentration, red=high concentration) and flow (yellow arrows, arrow length proportional to flow intensity). Each figure shows a steady inward flow of oxygen.
- figures S11-14: extracellular glucose concentration (color mapping, blue=low concentration, red=high concentration) and flow (yellow arrows, arrow length proportional to flow intensity). Initially the flow of extracellular glucose points only inward, but glucose diffusion is slow, and as the spheroid grows and dead cells in the core contract, an internal region with an outward flow of extracellular glucose slowly develops.
- figures S15-17: extracellular glutamine concentration (color mapping, blue=low concentration, red=high concentration) and flow (yellow arrows, arrow length proportional to flow intensity). Glutamine behaves much like glucose, and these snapshots show the glutamine flow before and after the development of the split flow.
- figure S18: lactate concentration (color mapping, blue=low concentration, red=high concentration) and flow (yellow arrows, arrow length proportional to flow intensity). Lactate always flows outward in the simulation: this is a single snapshot taken after both glucose and glutamine have developed their split flow regime.
- figures S19-21: velocity in the plane of the slice (yellow arrows, arrow length proportional to flow intensity). Cells in the core perform complex looping motions, while cells in the viable rim always push outward.

Short movies showing features of the same central slice are also available as additional supplementary information:

- S1: development of the necrotic core (red = live cells, black = dead cells).
- S2: flow of extracellular glucose (same coding as figures S11-14);
- S3: map of projected cell velocities (same coding as figures S19-21).

# References

[1] Chignola R, Milotti E (2005) A phenomenological approach to the simulation of metabolism and proliferation dynamics of large tumor cell populations. Phys. Biol. 2: 8-22.

[2] Chignola R, Del Fabbro A, Dalla Pellegrina C, Milotti E (2007) Ab initio phenomenological simulation of the growth of large tumor cell populations. Phys. Biol. 4: 114-133.

[3] Zetterberg A, Larsson O (1985) Kinetic analysis of regulatory events in G1 leading to proliferation or quiescence of Swiss 3T3 cells. Proc. Natl Acad. Sci. USA 82: 5365-5369.

[4] Milotti E, Del Fabbro A, Chignola R (2009) Numerical integration methods for large-scale biophysical simulations. Comp. Phys. Comm. 180: 2166-2174.

[5] Catlett NL, Weisman LS (2000) Divide and multiply: organelle partitioning in yeast. Curr. Opin. Cell Biol. 12: 509-516.

[6] Bergeland T, Widerberg J, Bakke O, Nordeng TW (2001) Mitotic partitioning of endosomes and lysosomes. Curr. Biol. 11: 644-651.

[7] Milotti E, Del Fabbro A, Dalla Pellegrina C, Chignola R (2008) Statistical approach to the analysis of cell desynchronization data. Physica A 387: 4204-4214.

[8] Johnson LV, Walsh ML, Chen LB (1980) Localization of mitochondria in living cells with rhodamine 123. Proc. Natl. Acad. Sci. USA 77: 990-994.

[9] Lang F, Waldegger S (1997) Regulating cell volume. Am. Sci. 85: 440-447.

[10] Wehner F, Olsen H, Tinel H, Kinne-Saffran E, Kinne RKH (2003) Cell volume regulation: osmolytes, osmolyte transport, and signal transduction. Rev. Physiol. Biochem. Pharmacol. 148: 1-80.

[11] Bortner CD, Cidlowski JA (2002) Apoptotic volume decrease and the incredible shrinking cell. Cell Death Diff. 9: 1307-1310.

[12] Newell K, Franchi A, Pouyssegur J, Tannock I (1993) Studies with glycolysis-deficient cells suggest that production of lactic acid is not the only cause of tumor acidity. Proc. Natl. Acad. Sci. USA 90: 1127-1131.

[13] Kaminskas E (1978) The pH-dependence of sugar transport and of glycolysis in cultured Ehrlich ascites-tumor cells. Biochem. J. 172: 453-459.

[14] Tildon JT, McKenna MC, Stevenson J, Couto R (1993) Transport of L-lactate by cultured rat brain astrocytes. Neurochem. Res. 18: 177-184.

[15] McKenna MC, Tildon JT, Stevenson JH, Hopkins IB, Huang X, Couto R (1998) Lactate transport by cortical synaptosomes from adult rat brain: characterization of kinetics and inhibitor specificity. Dev. Neurosci. 20: 300-310.

[16] Bröer A, Albers A, Setiawan I, Edwards RH, Chaudhry FA, Lang F, Wagner S, Bröer CA (2002) Regulation of the glutamine transporter SN1 by extracellular pH and intracellular sodium ions. J. Physiol. 539: 3-14.

[17] Ezhevsky SA, Ho A, Becker-Hapak M, Davis PK, Dowdy SF (2001) Differential regulation of retinoblastoma tumor suppressor protein by G1 cyclin-dependent kinases complexes in vivo. Mol. Cell Biol. 21: 4773-4784.

[18] Seville LL, Shah N, Westwell AD, Chan WC (2005) Modulation of pRb/E2F functions in the regulation of cell cycle and in cancer cell. Curr. Cancer. Drug Targets 5: 159-170.

[19] Chignola R, Del Fabbro A, Dalla Pellegrina C, Milotti E (2006) Thresholds, long delays and stability from generalized allosteric effect in protein networks. Physica A 371: 463-472.

[20] Milotti E, Del Fabbro A, Dalla Pellegrina C, Chignola R (2007) Dynamics of allosteric action in multisite protein modification. Physica A 379: 133-150.

[21] Chapman JD (2003) Single-hit mechanism of tumor cell killing by radiation. Int. J. Rad. Biol. 79: 71-81.

[22] The Delaunay triangulation and its dual, the Voronoi diagram, bears a strong resemblance to actual cellular structures, and for this reason it has been repeatedly used in models of cell clusters. In addition to standard references on these geometrical structures we include a few references to biological applications that are however just a small sample of the extended literature on this topic.

[23] de Berg M, Cheong O, van Kreveld M, Overmars M (2008) Computational Geometry: Algorithms and Applications. Berlin: Springer Verlag, 3rd edition.

[24] O’Rourke J (1996) Computational Geometry in C. New York: Cambridge Univ. Press, 2nd edition.

[25] Kansal AR, Torquato S, Harsh IV GR, Chiocca EA, Deisboeck TS (2000) Simulated brain tumor growth dynamics using a three-dimensional cellular automaton. J. Theor. Biol. 203: 367-382.

[26] Honda H, Tanemura M, Yoshida A (2000) Differentiation of wing epidermal scale cells in a butterfly under the lateral inhibition model – appearance of large cells in a polygonal pattern. Acta Biotheor. 48: 121-136.

[27] Beyer T, Meyer-Hermann M (2007) Modeling emergent tissue organization involving high-speed migrating cells in a flow equilibrium. Phys. Rev. E 76: 021929.

[28] Li X, Cristini V, Nie Q, Lowengrub JS (2007) Nonlinear three-dimensional simulation of solid tumor growth. Discr. Cont. Dyn. Sys. – Ser. B 7: 581-607.

[29] Gershenfeld N (1999) The nature of mathematical modeling. New York: Cambridge Univ. Press.

[30] See, e.g., Press WH, Teulkosky SA, Vetterling WT, Flannery BP (2007) Numerical Recipes: The Art of Scientific Computing. New York: Cambridge Univ. Press, 3rd ed.

[31] Verdier C (2003) Rheological properties of living materials. From cells to tissues. J. Theor. Med. 5: 67-91.

[32] Discher DE, Boal DH, Boey SK (1988) Simulations of the erythrocyte cytoskeleton at large deformation. II. Micropipette aspiration. Biophys. J. 75: 1584-1597.

[33] Yeung A, Evans E (1989) Cortical shell-liquid core model for passive flow of liquid-like spherical cells into micropipets. Biophys. J. 56: 139-149.

[34] Hochmuth RM, Ting-Beall HP, Beaty BB, Needham D, Tran-Son-Tay R (1993) Viscosity of passive human neutrophils undergoing small deformations. Biophys. J. 64: 1596-1601.

[35] Heinrich V, Ritchie K, Mohandas N, Evans E (2001) Elastic thickness compressibility of the red cell membrane. Biophys. J. 81: 1452-1463.

[36] Bausch AR, Möller W, Sackmann E (1999) Measurement of local viscoelasticity and forces in living cells by magnetic tweezers. Biophys. J. 76: 573-579.

[37] Sleep J, Wilson D, Simmons R, Gratzer W (1999) Elasticity of the red cell membrane and its relation to hemolytic disorders: an optical tweezers study. Biophys. J. 77: 3085-3095.

[38] Radmacher M (1997) Measuring the elastic properties of biological samples with the AFM. IEEE Eng. Med. Biol. Mag. 16: 47-57.

[39] A-Hassan E (1998) *et al.* Relative microelastic mapping of living cells by atomic force microscopy. Biophys. J. 74: 1564-1578.

[40] Takeuchi M, Miyamoto H, Sako Y, Komizu H, Ksumi A (1998) Structure of the erythrocyte membrane skeleton as observed by atomic force microscopy. Biophys. J. 74: 2171-2183.

[41] Lekka M et al (1999), Local elastic properties of cells studied by SFM. Appl. Surf. Sci. 141: 345-349.

[42] Lekka M et al (1999), Elasticity of normal and cancerous human bladder cells studied by scanning force microscopy. Eur. Biophys. J. 28: 312-316.

[43] Sen S, Subramanian S, Discher DE (2005) Indentation and adhesive probing of a cell membrane with AFM: theoretical model and experiments. Biophys. J. 89: 3203-3213.

[44] Rosenbluth MJ, Lam WA, Fletcher DA (2006) Force microscopy of nonadherent cells: A comparison of leukemia cell deformability. Biophys. J. 90: 2994-3003.

[45] Roca-Cusachs P et al (2006) Rheology of passive and adhesion-activated neutrophils probed by atomic force microscopy. Biophys. J. 91: 3508-3518.

[46] Fukumura D, Jain RK (2007) Tumor microenvironment abnormalities: causes, consequences, and strategies to normalize. J. Cell. Biochem. 101: 937-949.

[47] Palsson E (2001) A three-dimensional model of cell movement in multicellular systems. Future Gen. Comp. Sy. 17: 835-852.

[48] Galbusera F, Cioffi M, Raimondi MT (2008) An in silico bioreactor for simulating laboratory experiments in tissue engineering. Biomed. Microdev. 10: 547-554.

[49] Wei C, Lintilhac PM, Tanguay JJ (2001) An insight into cell elasticity and load-bearing ability. Measurement and theory. Plant Physiol. 126: 1120-1138.

[50] Galle J, Loeffler M, Drasdo D (2005) Modeling the effect of deregulated proliferation and apoptosis on the growth dynamics of epithelial cell populations in vitro. Biophys. J. 88: 62-75.

[51] Zhu C (2000) Kinetics and mechanics of cell adhesion. J. Biomech. 33: 23-33.

[52] Bell G, Dembo M, Bongrand P (1984) Cell adhesion. Competition between nonspecific repulsion and specific bonding. Biophys. J. 45: 1051-1064.

[53] Chandrasekhar S (1943) Stochastic problems in physics and astronomy. Rev. Mod. Phys. 15: 1-89.

[54] Flekkøy EG, Coveney PV, De Fabritiis G (2000) Foundations of dissipative particle dynamics. Phys. Rev. E 62: 2140-2157.

[55] Dzwinel W, Yuen DA, Boryczko K (2002) Mesoscopic dynamics of colloids simulated with dissipative particle dynamics and fluid particle model. J. Mol. Model. 8: 33-43.

[56] Butcher JC (2008) Numerical methods for ordinary differential equations. Chichester: Wiley.

[57] Ascher U, Ruuth S, Spiteri RJ (1997) Implicit-explicit Runge-Kutta methods for time dependent partial differential equations. Appl. Numer. Math. 25: 151-167.

[58] Maleki A, Kjøniksen A-L, Nyström B (2007) Anomalous viscosity behavior in aqueous solutions of hyaluronic acid. Polym. Bull. 59: 217-226.

[59] Newman S, Cloître M, Allain C, Forgacs G, Beysens D (1997) Viscosity and elasticity during collagen assembly in vitro: relevance to matrix-driven translocation. Biopolymers 41: 337-347.

[60] Regina A, Roux F, Revest PA (1997) Glucose transport in immortalized rat brain capillary endothelial cells in vitro: transport activity and GLUT1 expression. Biochim. Biophys. Acta 1335: 135-143.

[61] Heimberg H et al (1996) The glucose sensor protein glucokinase is expressed in glucagon-producing alfa-cells. Proc. Natl. Acad. Sci. USA 93: 7036-7041.

[62] Mazurek S, Zwerschke W, Jansen-Dürr P, Eigenbrodt E (2001) Effects of the human papilloma virus HPV-16 E7 oncoprotein on glycolysis and glutaminolysis: role of pyruvate kinase type M2 and the glycolytic-enzyme complex. Biochem. J. 356: 247-256.

[63] Levintow L, Eagle H, Piez KA (1955) The role of glutamine in protein biosynthesis in tissue culture. J. Biol. Chem. 215: 441-460.

[64] Salzman NP, Eagle H, Sebring ED (1958) The utilization of glutamine, glutamic acid, and ammonia for the biosynthesis of nucleic acid bases in mammalian cell cultures. J. Biol. Chem. 230: 1001-1012.

[65] Voet D, Voet J (2002) Biochemistry New York: Wiley, 2nd ed.

[66] Robin ED, Wong R (1988) Mitochondrial DNA molecules and virtual number of mitochindria per cell in mammalian cells. J. Cell Physiol. 136: 507-513.

[67] James TW, Bohman R (1981) Proliferation of mitochondria during the cell cycle of the human cell line (HL-60). J. Cell Biol. 89: 256-260.

[68] Masafumi W, Wang HS, Okada A (2002) Characterization of L-glutamine transport by human neuroblastoma cell line. Am. J. Physiol. Cell. Physiol. 282: C1246-C1253.

[69] Rodriguez-Enriquez S, Torres-Marquez ME, Moreno-Sanchez R (2000) Substrate oxydation and ATP supply in AS-300 hepatoma cells. Arch. Biochem. Biophys. 375: 21-30.

[70] Porwol T, Merten E, Opitz N, Acker H (1996) Three-dimensional imaging of rodhamine 123 fluorescence distribution in human melanoma cells by means of confocal laser scanning microscopy. Acta Anat. (Basel) 157: 116-125.

[71] Halestrap AP, Price NT (1999) The proton-linked monocarboxylate transporter (MCT) family: structure, function and regulation. Biochem. J. 343: 281-299.

[72] Spencer TL, Lehninger AL (1976) L-lactate transport in Erlich ascites-tumour cells. Biochem. J. 154: 405-414.

[73] von Grumbckow L, Elsner P, Hellsten Y, Quistroff B, Juel C (1999) Kinetics of lactate and pyruvate transport in cultured rat myotubes. Biochim. Biophys. Acta 1417: 267-275.

[74] Clare PM et al (2001) The cyclin-dependent kinases cdk2 and cdk5 act by a random anticooperative kinetic mechanism. J. Biol. Chem. 276: 48292-48295.

[75] Bertoni-Freddari C et al (2007) Preservation of mitochondrial volume homeostasis at the early stages of age-related synaptic deterioration. Ann. N.Y. Acad. Sci. 1096: 138-146.

[76] a very readable introductory text on elasticity theory is ch. 38 in Feynman RP, Leighton RB, Sands M (1964) The Feynman Lectures on Physics, vol. 2, Reading: Addison-Wesley.

[77] Ng CE, Inch WR (1978) Comparison of the densities of clonogenic cells from EMT6 fibrosarcoma monolayer cultures, multicell spheroids, and solid tumors in ficoll density gradients. J. Natl. Cancer Inst. 60: 1017-1022.

[78] Nilsson B, Olofsson T (1985) Density distribution of chronic myeloid leukaemia and normal colony-forming cells in diffusion chambers (CFU-D) and agar (CFU-GM). Scand. J. Haematol. 34: 317-322.

[79] Evans E, Yeung A (1989) Apparent viscosity and cortical tension of blood granulocytes determined by micropipet aspiration. Biophys. J. 56: 151-160.

[80] Sloane NJA (1998) The Sphere Packing Problem. Documenta Mathematika, III: 387-396.

[81] Sloane NJA (1998) Kepler's Conjecture Confirmed. Nature 395: 435-436.

[82] Jain RK (1987) Transport of Molecules in the Tumor Interstitium: A Review. Cancer Res. 47: 3039-3051.

[83] Krol A, Maresca J, Dewhirst MW, Yuan F (1999) Available Volume Fraction of Macromolecules in the Extravascular Space of a Fibrosarcoma: Implications for Drug Delivery. Cancer Res. 59: 4136–4141.

[84] Kim YR, Savellano MD, Savellano DH, Weissleder R, Bogdanov Jr. A (2004) Measurement of Tumor Interstitial Volume Fraction: Method and Implication for Drug Delivery, Magnetic Resonance in Medicine 52: 485–494.

[85] Casciari JJ, Sotirchos SV, Sutherland RM (1988) Glucose Diffusivity in Multicellular Tumor Spheroids, Cancer Res. 48: 3905–3909.

[86] Nielsen TA, DiGregorio DA, Angus Silver R (2004) Glutamate Mobility Reveals the Mechanism Underlying Slow-Rising AMPAR EPSCs and the Diffusion Coefficient in the Synaptic Cleft. Neuron 42: 757–771.

[87] Vega C, Poitry-Yamate CL, Jirounek P, Tsacopoulos M, Coles JA (1998) Lactate Is Released and Taken Up by Isolated Rabbit Vagus Nerve During Aerobic Metabolism, *J.* Neurochem. 71: 330-337.

[88] Dwyer RA (1989) Higher-dimensional Voronoi diagrams in linear expected time. In Proc. 5th Annu. ACM Sympos. Comput. Geom., 326-333.

[89] Dwyer RA (1991) Higher-dimensional Voronoi diagrams in linear expected time. Discr. and Comp. Geom. 6: 343-367.

[90] Guibas LJ, Knuth DE, Sharir M (1992), Randomized incremental construction of Delaunay and Voronoi diagram. Algorithmica 7: 381-413.

[91] We use the randomized incremental construction of reference [90] as implemented in the computational geometry library CGAL ([http://www.cgal.org](http://www.cgal.org/)).

1. Notice also that a term *ConsATP_4_* is missing: the name is reserved to a class of processes not yet included in the program. [↑](#footnote-ref-2)
