# Supplementary figures and images for: Emergent Properties of Tumor Microenvironment in a Real-Life Model of Multicell Tumor Spheroids
Source: PLoS One. 2010 Nov 30;5(11):e13942. doi: 10.1371/journal.pone.0013942 (PMC2994713; doi:10.1371/journal.pone.0013942)

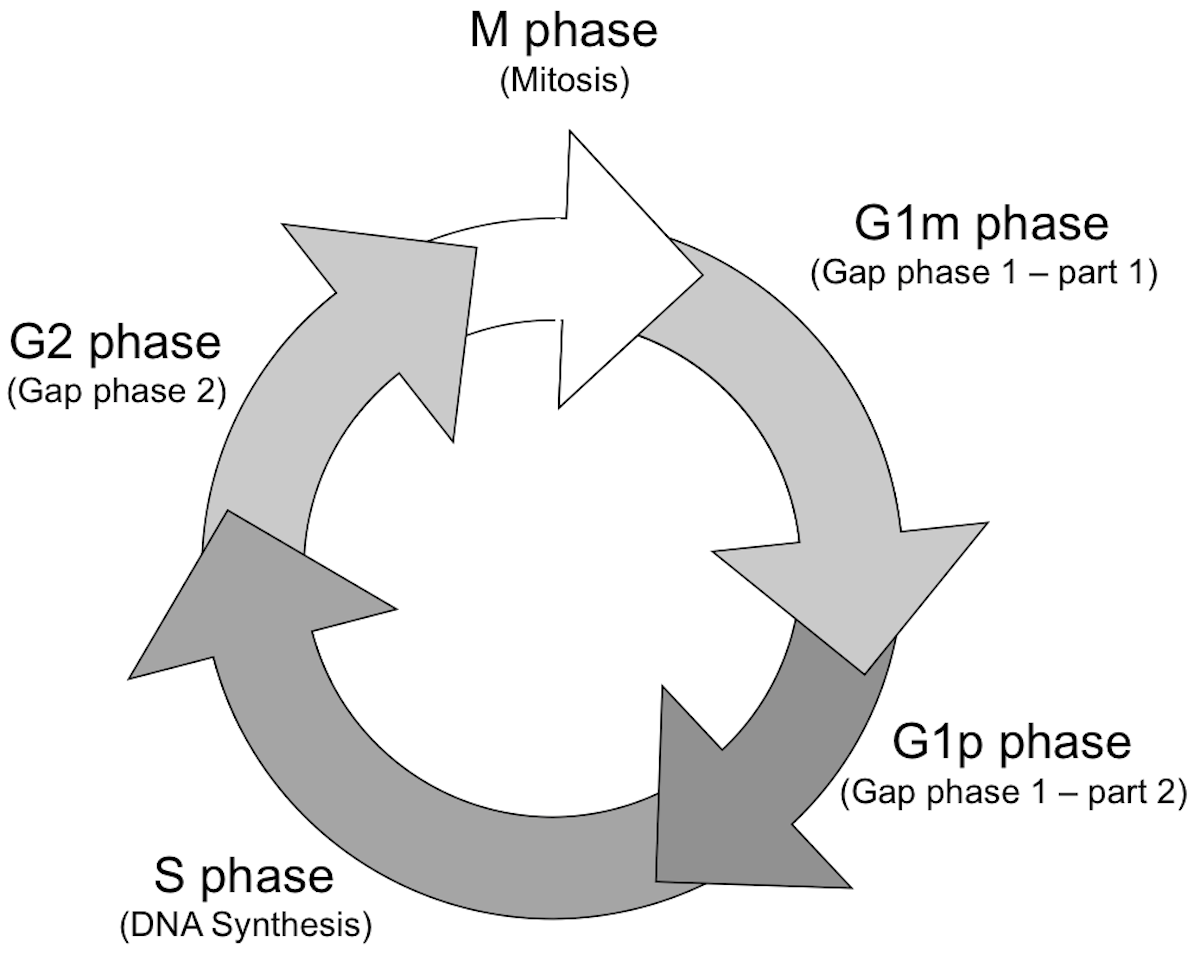

Supplement: Figure S1 — Sketch of the cell phases included in the simulation program. The arrow lengths suggest the relative duration of each phase. Phase G1 is divided in two parts: an initial subphase where cells are sensitive to variations in the environmental nutrient concentration (G1m) and a later subphase where cells are insensitive to deprivation of nutrients (G1p); in between these subphases there is an energetic checkpoint [1]–[3]. (0.19 MB TIF) [file pone.0013942.s002.tif]

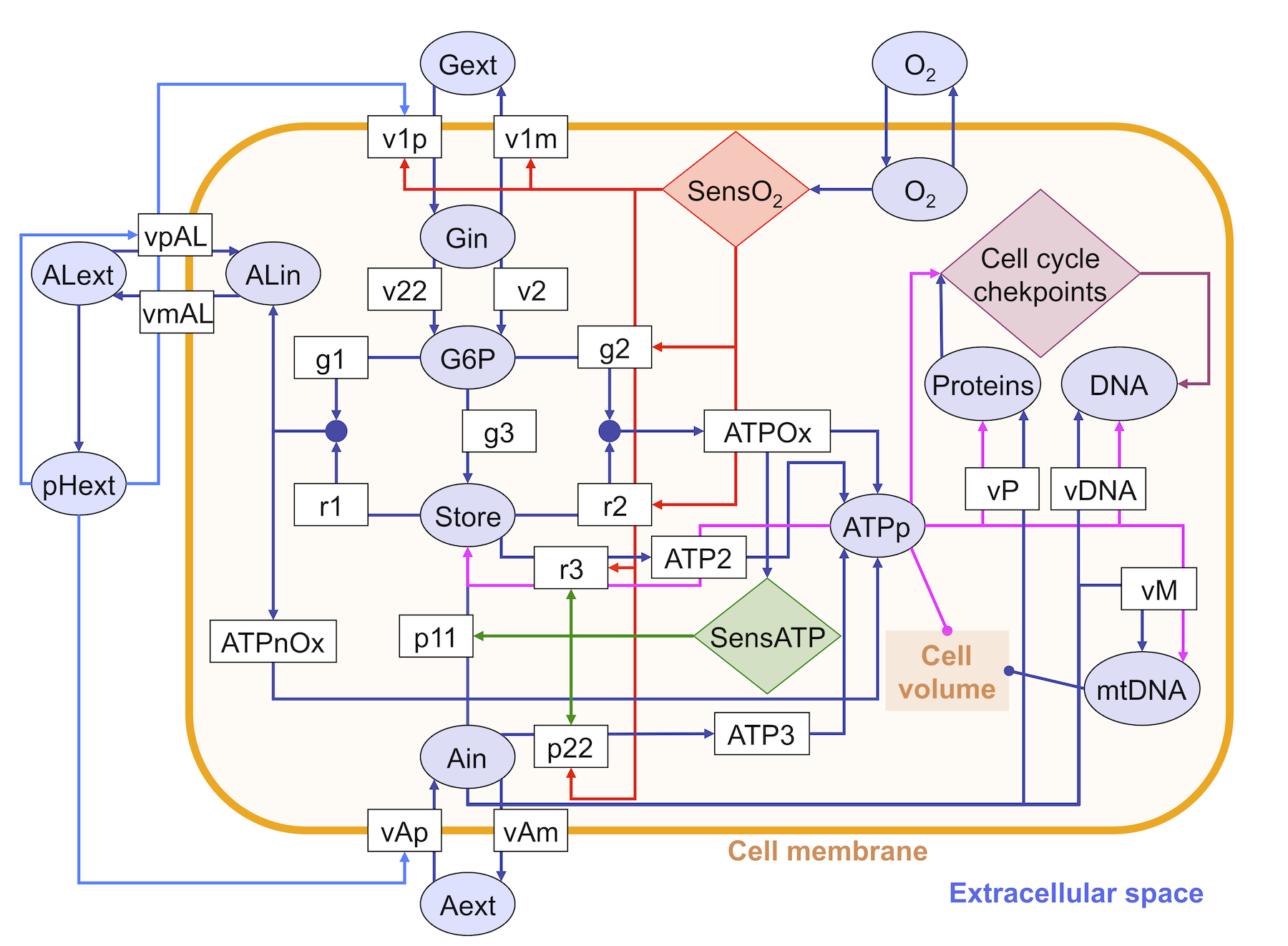

Supplement: Figure S2 — Sketch of the metabolic network. Variables within circles represent molecular species and are expressed in units of concentration or mass. Non-obvious symbols are as follows (the suffixes ext and int denote, respectively, extracellular and intracellular variables): G = glucose, G6P = glucose-6-phosphate, STORE = glucose stored in the form of glycogen, AL = lactic acid, A = glutamine, ATPp = pool of ATP molecules, DNA = nuclear mass of DNA (normalized to 1 for the whole genome), mtDNA = mitochondrial DNA. Rates are represented by squares. The red circuit represents the oxygen sensor, whereas the green circuit represents the ATP sensor [1], [2]. “Cell cycle checkpoints” denotes the molecular circuit of cell cycle control that has been modeled on the basis of previous studies on the dynamics of the allosteric effect [19], [20]. The biological foundations of this simplified metabolic network have been given in references 1 and 2. Recent improvements with respect to our previous model include: internalization rates of glucose, glutamine and lactate are sensitive to extracellular pH and this dependence is now described by smoothed functions (see the text for details); synthesis of cellular proteins, nuclear DNA and mitochondrial DNA are now described by double-substrate Michaelis-Menten chemical reactions to take into account the dependence of protein and nucleic acid biosynthesis on glutamine (which stands phenomenologically for the wider class of aminoacids) and ATP availability. (0.77 MB TIF) [file pone.0013942.s003.tif]

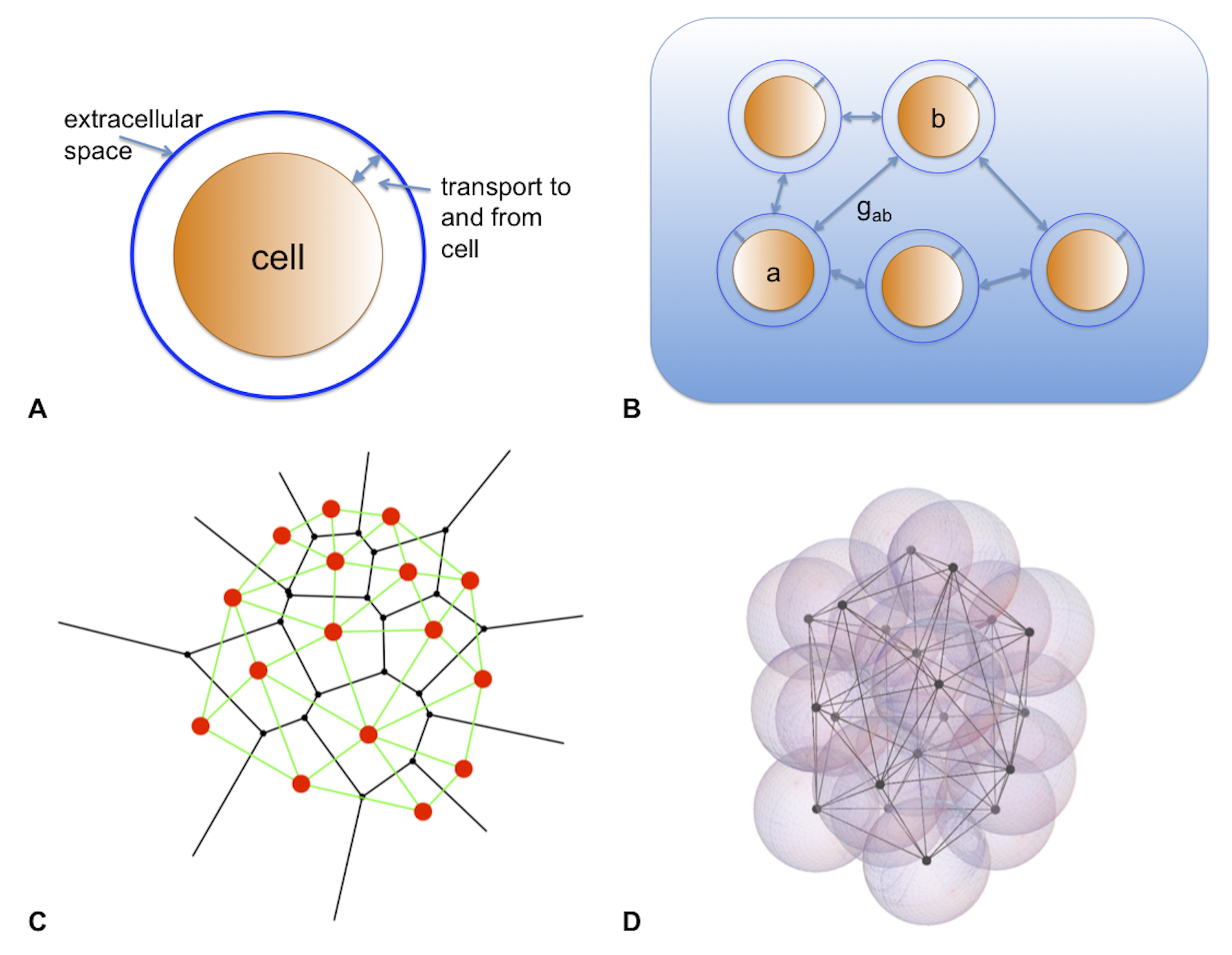

Supplement: Figure S3 — The geometry and topology of diffusion. a) Most substances are carried into and out of the cell by facilitated diffusion and there is an active mass exchange between cell and extracellular space. Each cell in the simulation program has its own extracellular space. b) Extracellular spaces are interconnected and there is a diffusion flow through the network of connections. c) The network of interconnected spaces is defined by a Delaunay triangulation. In this 2D representation, for any red dot we can define a Voronoy cell, i.e., the set of points in the plane that are closer to the given dot than to any other dot in the set. The dual structure is the Delaunay triangulation (Voronoy cells are black and Delaunay links are green). There is a Delaunay link between any two dots only if the respective Voronoy cells touch each other, therefore we can use the Delaunay triangulation to define proximity. This enables us to set up a discretized version of diffusion between extracellular spaces, like in b). In addition to the topology of contacts between cells we also keep into account geometry: gab in part b) is a geometric factor that modulates diffusion. d) The actual simulation is in 3D: here the Delaunay triangulation of a small cluster of cell centers shows up in transparency. (1.37 MB TIF) [file pone.0013942.s004.tif]

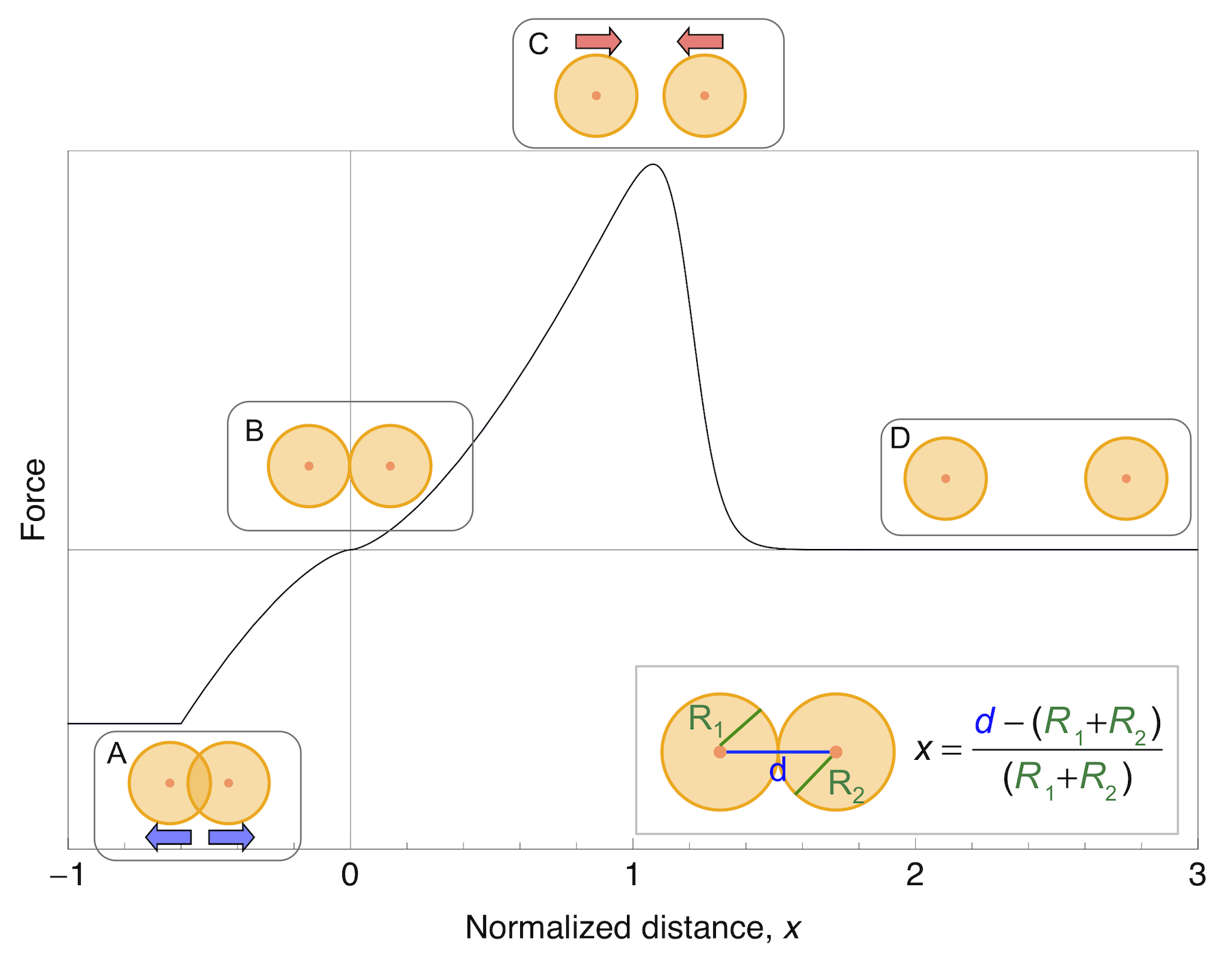

Supplement: Figure S4 — Pictorial representation of the interaction force between two cells. The solid curve shows qualitatively the behavior of the interaction force, while the insects depict the corresponding situations (A. cells are compressed against each other, force is repulsive; B. cells are in contact, the total force vanishes; C. cell centers are slightly apart, force is attractive because of adhesive molecules on the cells' membranes; D. cells are no longer in contact, the total force vanishes again). The inset on the right corner shows the definitions of the basic geometric variables. (0.36 MB TIF) [file pone.0013942.s005.tif]

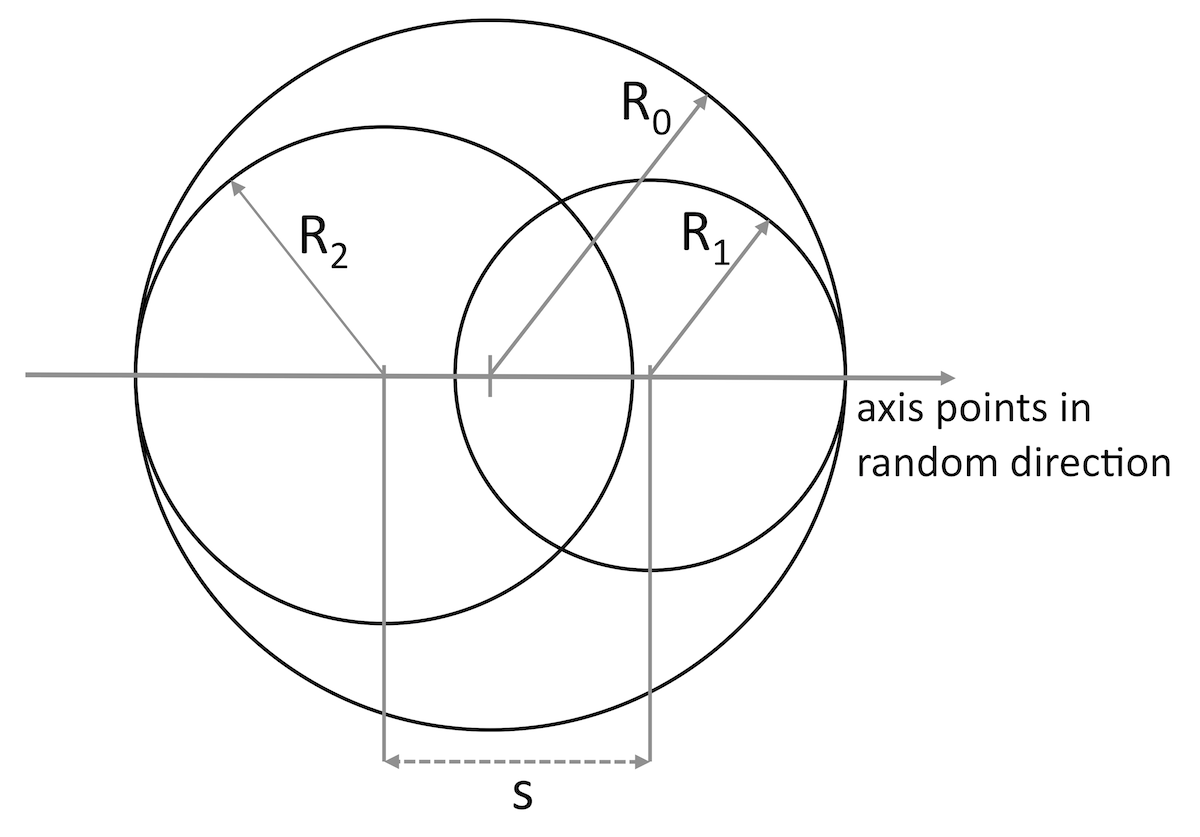

Supplement: Figure S5 — The geometry of mitosis. R0 is the radius of the initial cells, while R1 and R2 are the radii of the daughter cells: because of random asymmetries during mitosis, the daughter cells usually have different sizes. The program places the two daughter cells inside the region initially occupied by the mother, and the axis connecting the centers points in a random direction. This forces the two cells to push one against the other, and as cells separate the axis rotates, so that the new configuration fits the positions of neighboring cells. The cells' centers are separated by a distance which is roughly 0.4 R0. (0.16 MB TIF) [file pone.0013942.s006.tif]

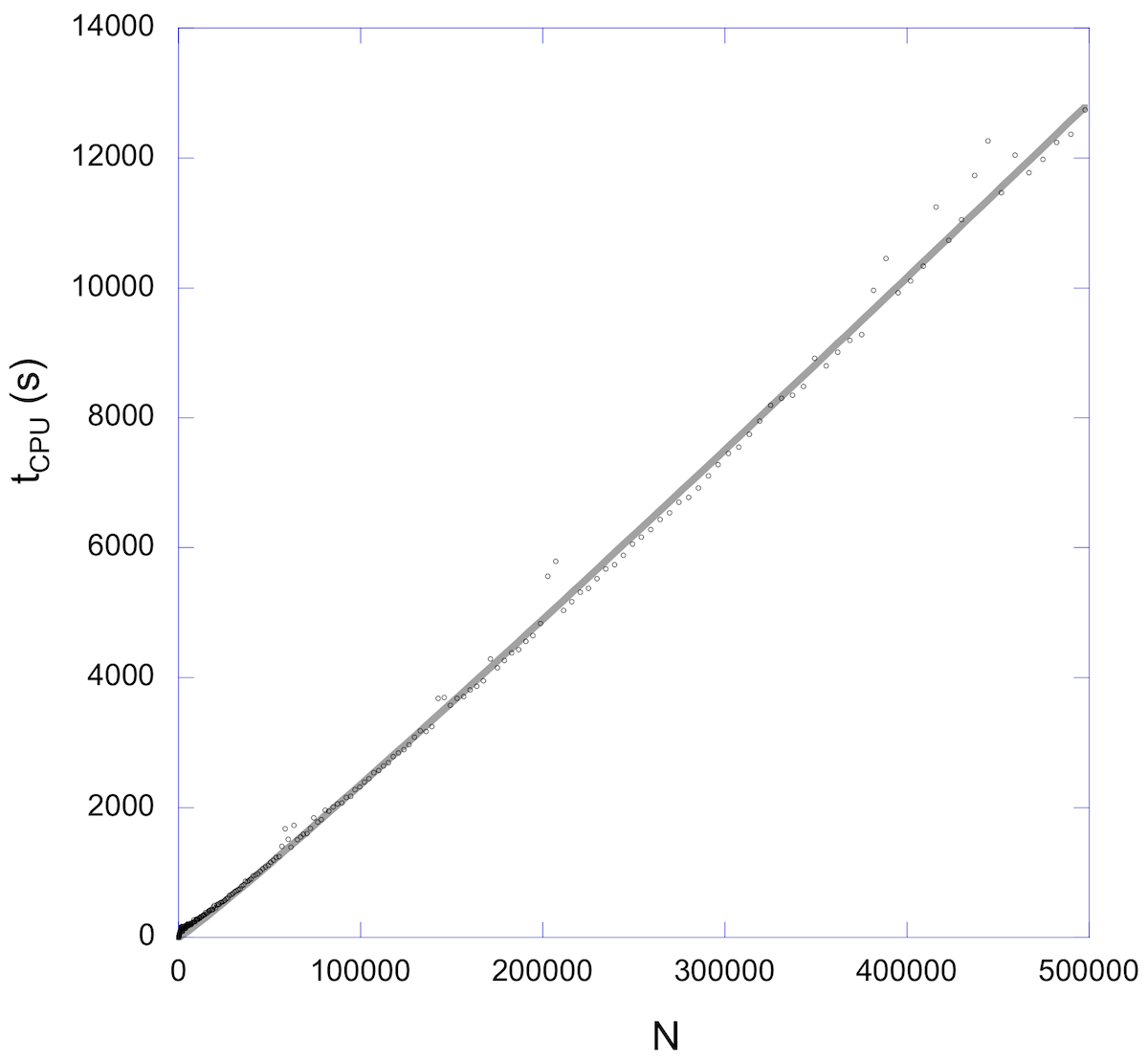

Supplement: Figure S6 — CPU time needed to simulate 1 hour, vs. the number of cells in the spheroid. In this run, the precision of the solution of the global diffusion transport and metabolism problem is fixed at 1% and the timestep is 50 s (so that tCPU is actually the CPU time needed to simulate 72 elementary timesteps). The solid curve shows the fit (S.69) in Text S1. (0.15 MB TIF) [file pone.0013942.s007.tif]

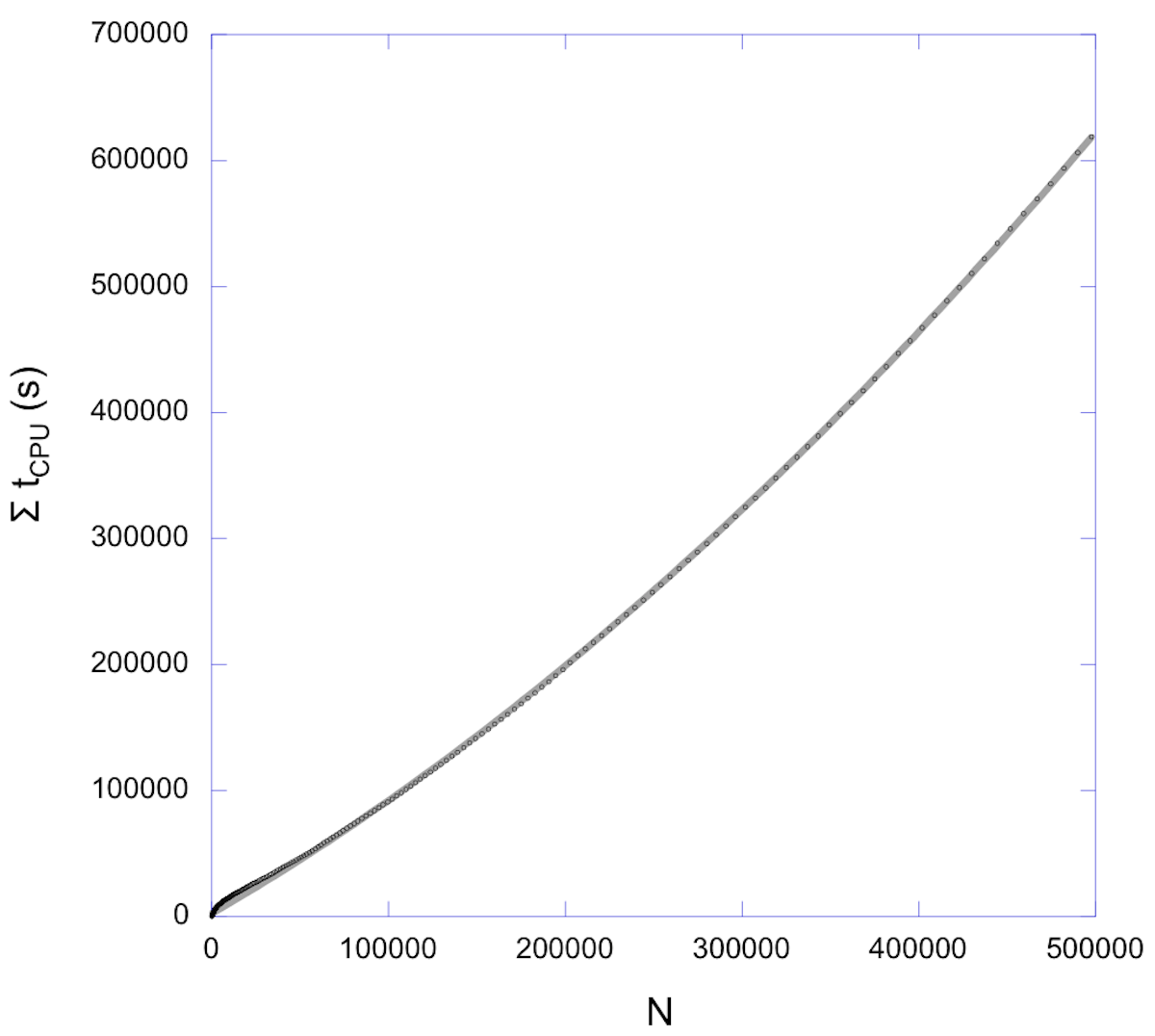

Supplement: Figure S7 — Total CPU time vs. N. This figure shows the total CPU time vs. N in the same run as figure S6. The solid curve is a simple fit with a quadratic polynomial function. (0.14 MB TIF) [file pone.0013942.s008.tif]

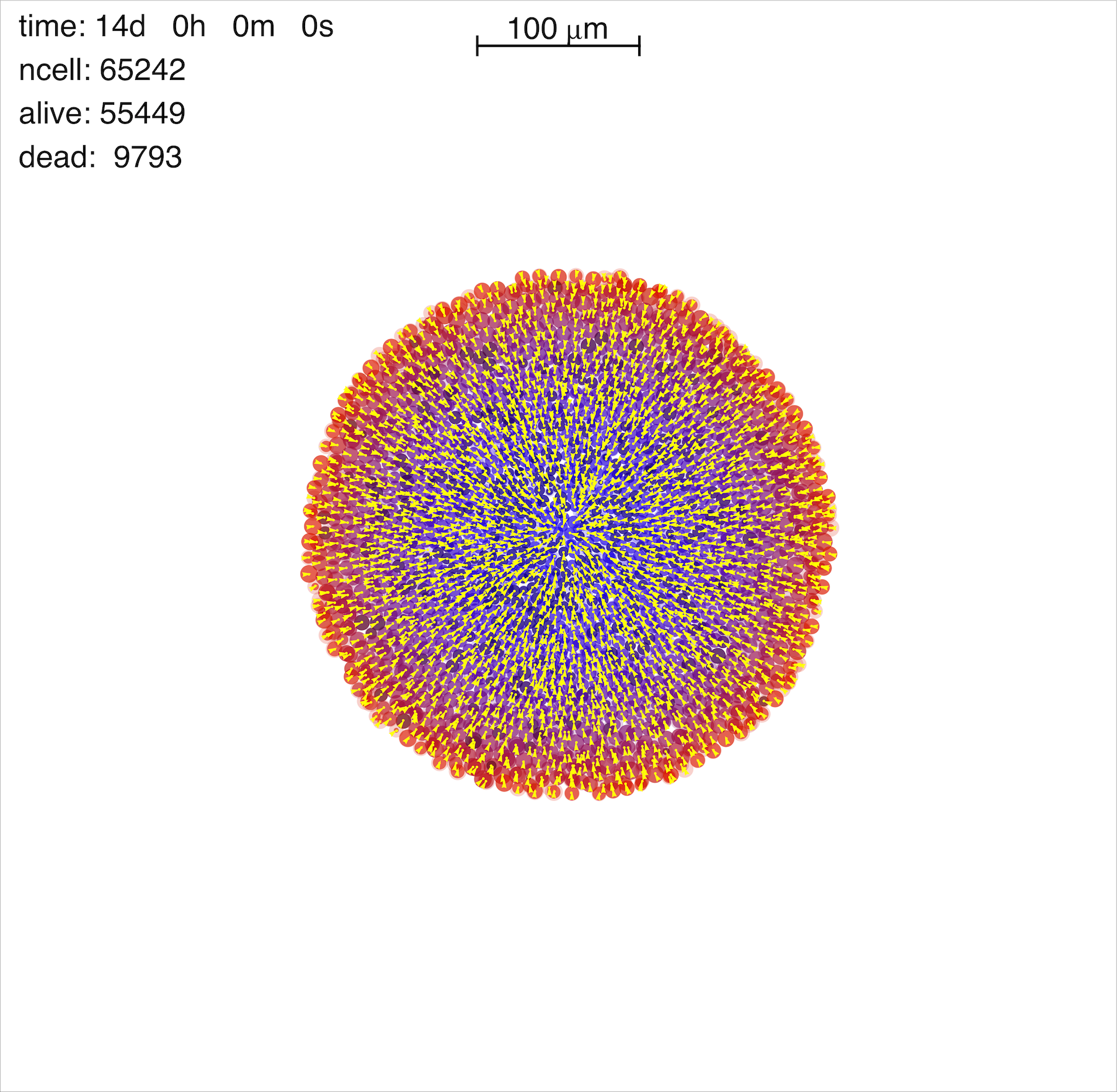

Supplement: Figure S8 — Oxygen concentration and flow at simulated time = 14 days. Cells are coloured to show the oxygen concentration (color mapping, blue = low concentration, red = high concentration), while the yellow arrows show the oxygen flow (arrow length proportional to flow intensity). (2.06 MB TIF) [file pone.0013942.s009.tif]

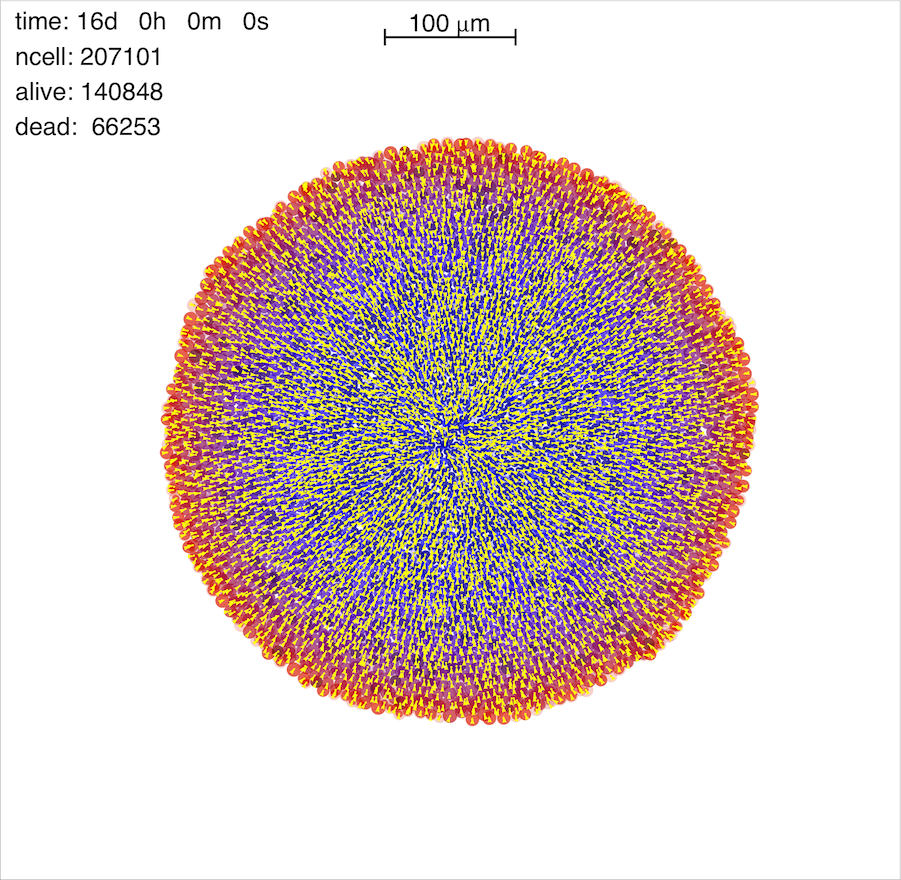

Supplement: Figure S9 — Oxygen concentration and flow at simulated time = 16 days. Cells are coloured to show the oxygen concentration (color mapping, blue = low concentration, red = high concentration), while the yellow arrows show the oxygen flow (arrow length proportional to flow intensity). (1.14 MB TIF) [file pone.0013942.s010.tif]

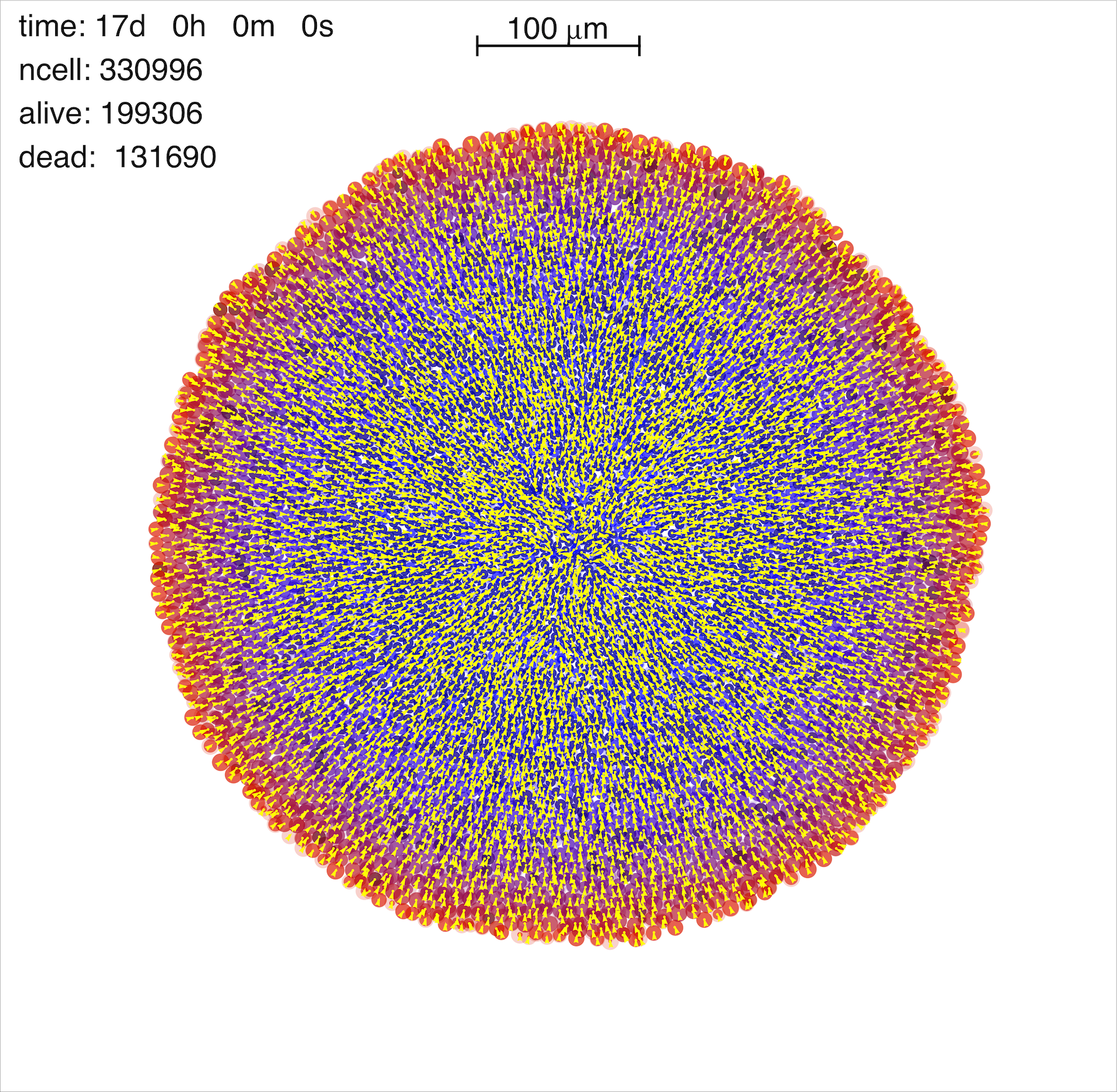

Supplement: Figure S10 — Oxygen concentration and flow at simulated time = 17 days. Cells are coloured to show the oxygen concentration (color mapping, blue = low concentration, red = high concentration), while the yellow arrows show the oxygen flow (arrow length proportional to flow intensity). (4.88 MB TIF) [file pone.0013942.s011.tif]

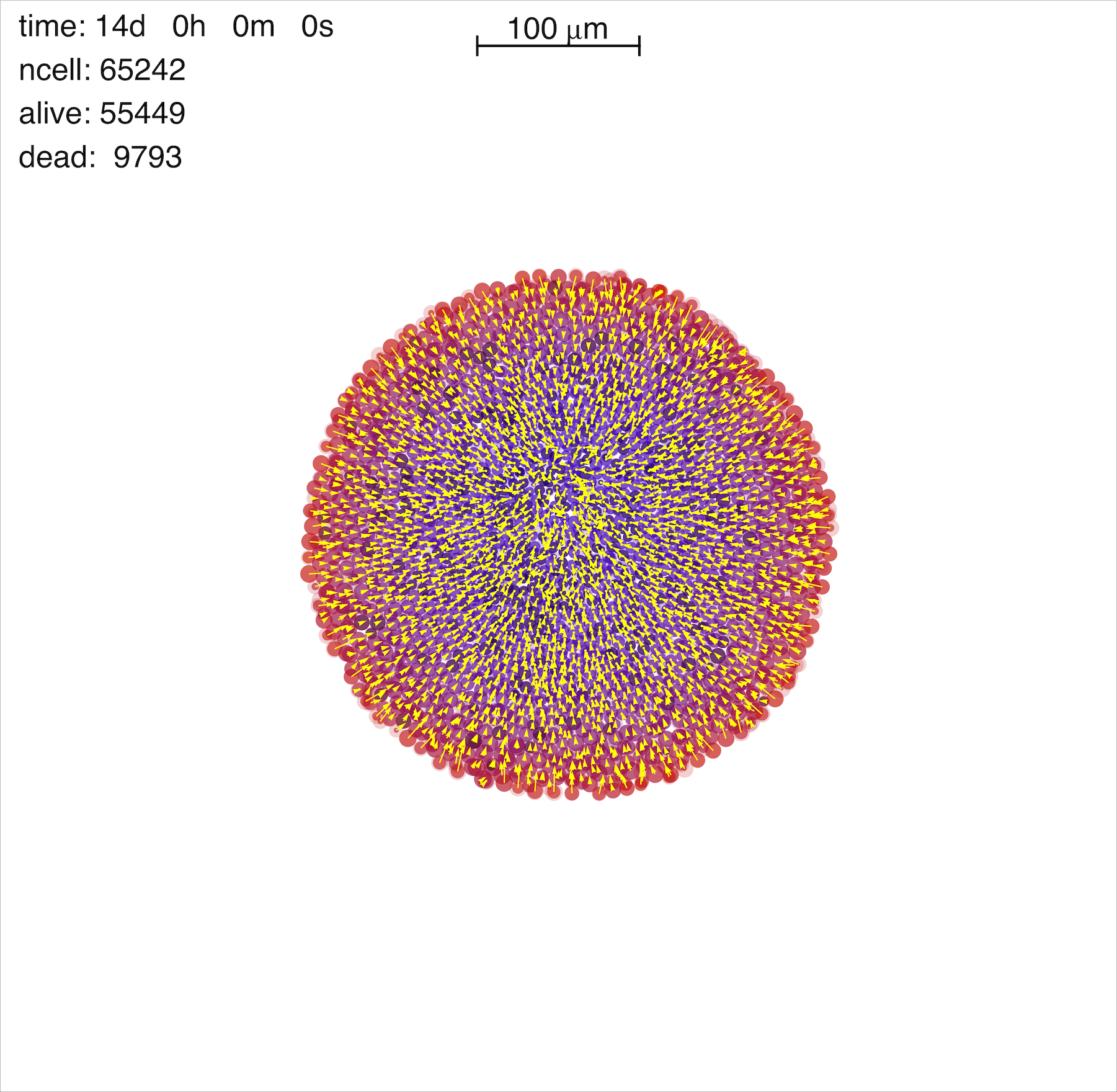

Supplement: Figure S11 — Extracellular glucose concentration and flow at simulated time = 14 days. Cells are coloured to show the concentration of extracellular glucose (color mapping, blue = low concentration, red = high concentration), while the yellow arrows show the flow of extracellular glucose (arrow length proportional to flow intensity). (2.05 MB TIF) [file pone.0013942.s012.tif]

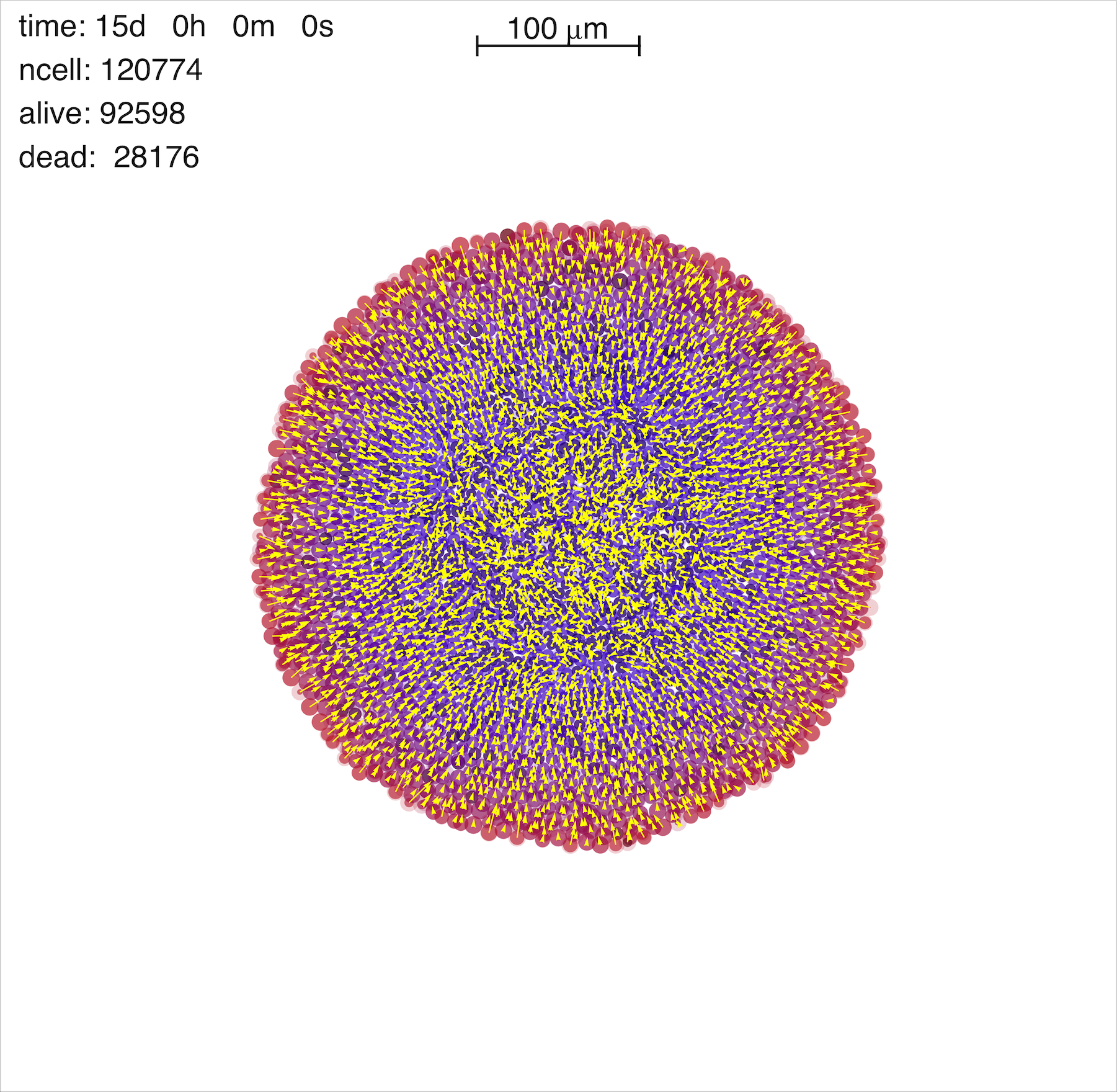

Supplement: Figure S12 — Extracellular glucose concentration and flow at simulated time = 15 days. Cells are coloured to show the concentration of extracellular glucose (color mapping, blue = low concentration, red = high concentration), while the yellow arrows show the flow of extracellular glucose (arrow length proportional to flow intensity). (2.88 MB TIF) [file pone.0013942.s013.tif]

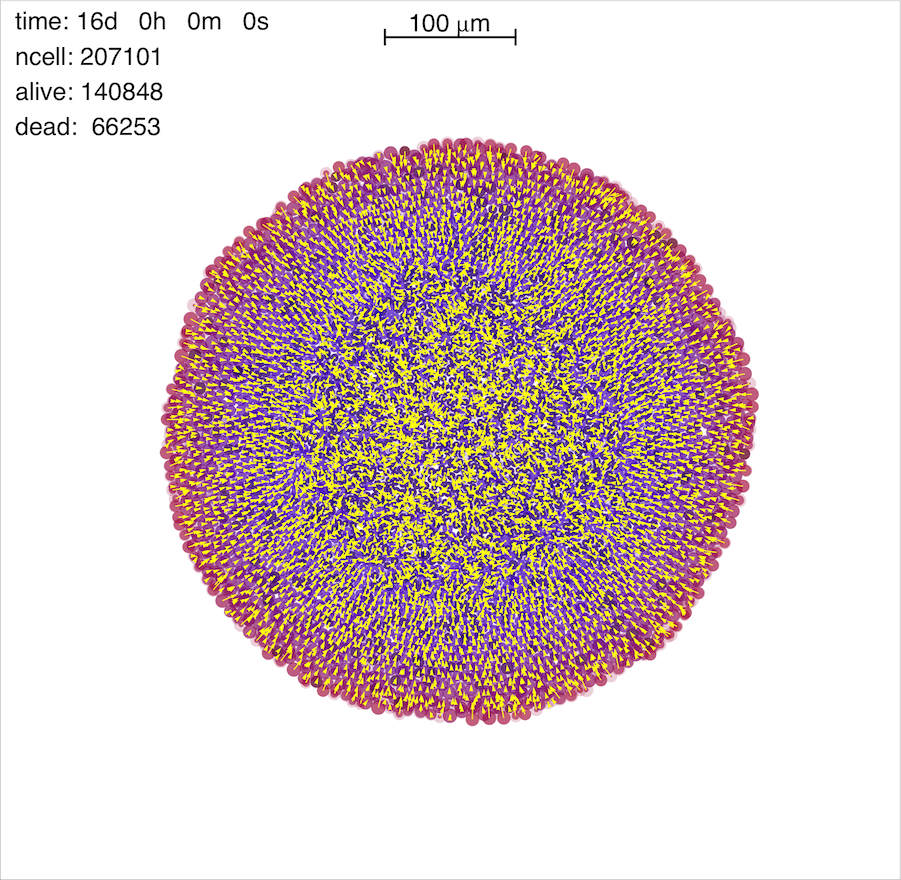

Supplement: Figure S13 — Extracellular glucose concentration and flow at simulated time = 16 days. Cells are coloured to show the concentration of extracellular glucose (color mapping, blue = low concentration, red = high concentration), while the yellow arrows show the flow of extracellular glucose (arrow length proportional to flow intensity). (1.15 MB TIF) [file pone.0013942.s014.tif]

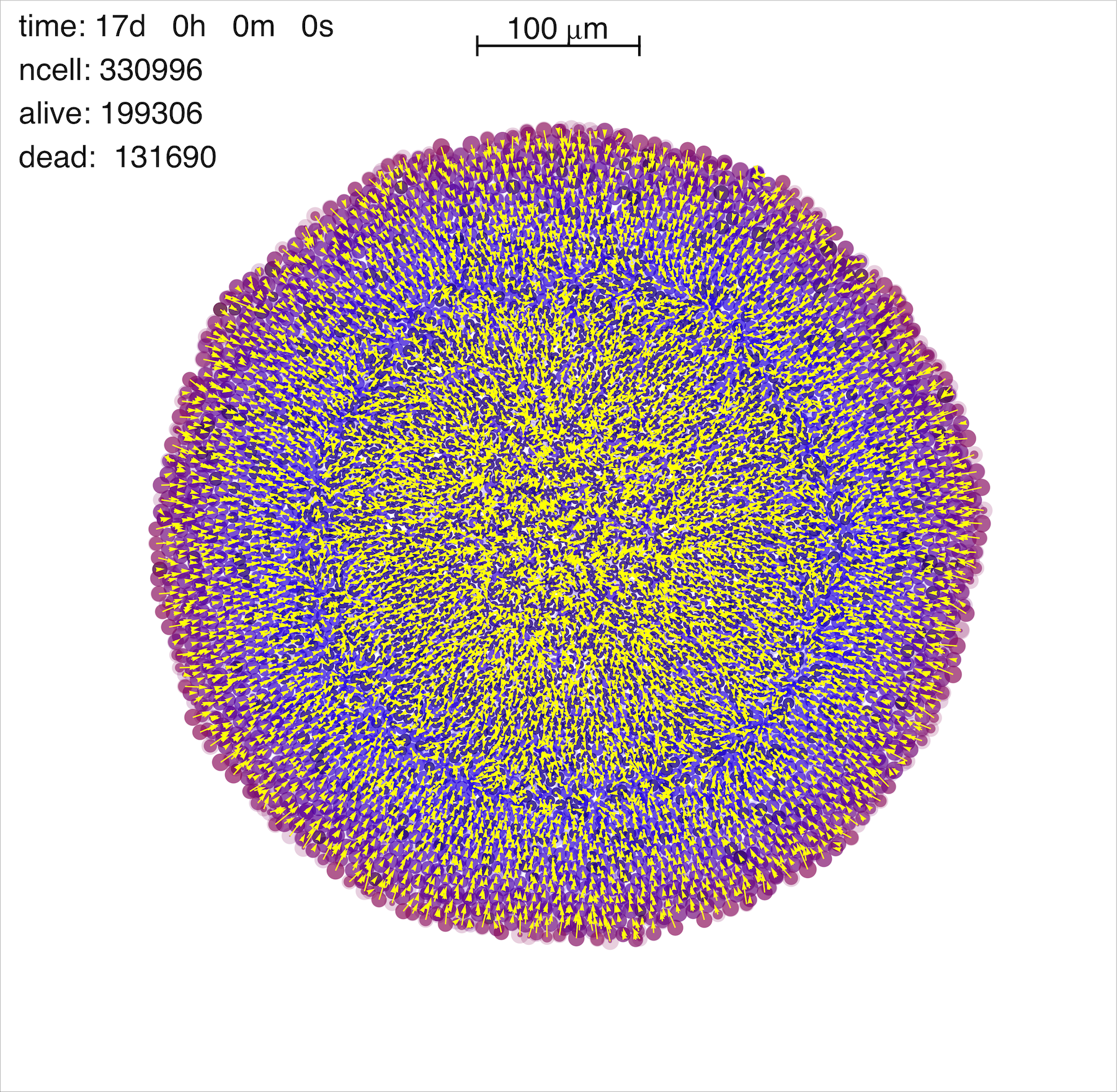

Supplement: Figure S14 — Extracellular glucose concentration and flow at simulated time = 17 days. Cells are coloured to show the concentration of extracellular glucose (color mapping, blue = low concentration, red = high concentration), while the yellow arrows show the flow of extracellular glucose (arrow length proportional to flow intensity). (4.97 MB TIF) [file pone.0013942.s015.tif]

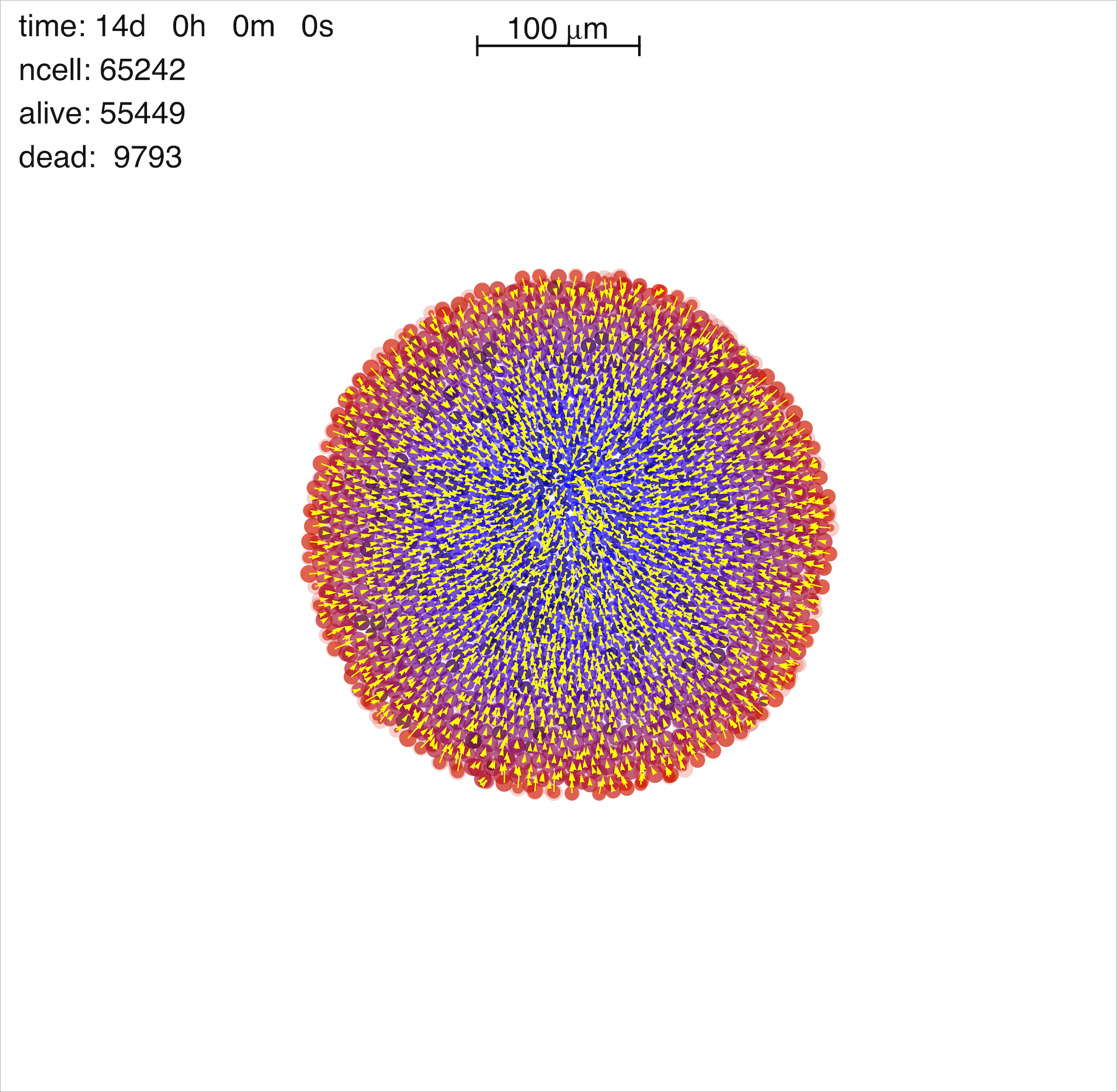

Supplement: Figure S15 — Extracellular glutamine concentration and flow at simulated time = 14 days. Cells are coloured to show the concentration of extracellular glutamine (color mapping, blue = low concentration, red = high concentration), while the yellow arrows show the flow of extracellular glutamine (arrow length proportional to flow intensity). (2.06 MB TIF) [file pone.0013942.s016.tif]

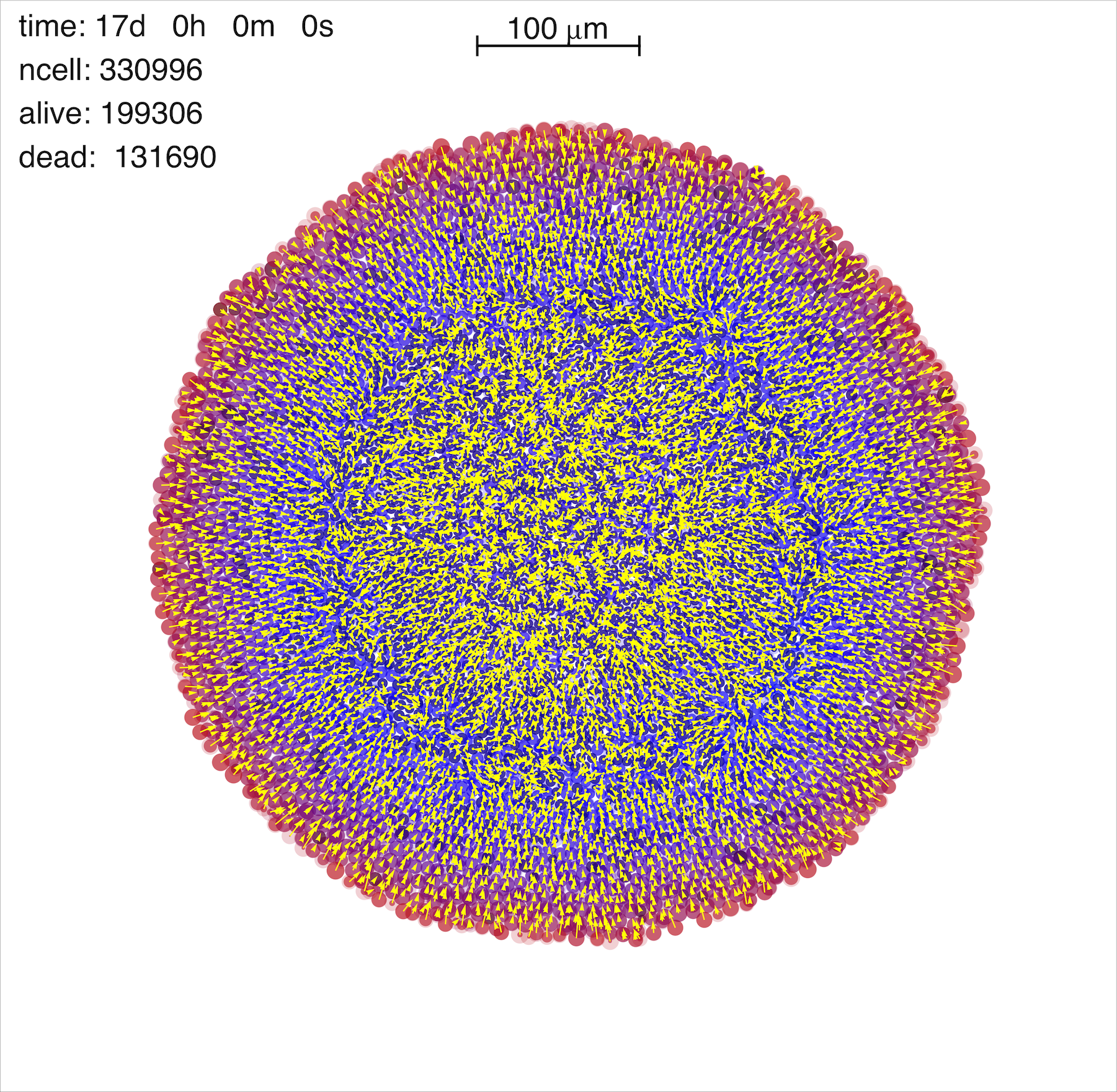

Supplement: Figure S16 — Extracellular glutamine concentration and flow at simulated time = 17 days. Cells are coloured to show the concentration of extracellular glutamine (color mapping, blue = low concentration, red = high concentration), while the yellow arrows show the flow of extracellular glutamine (arrow length proportional to flow intensity). (4.94 MB TIF) [file pone.0013942.s017.tif]

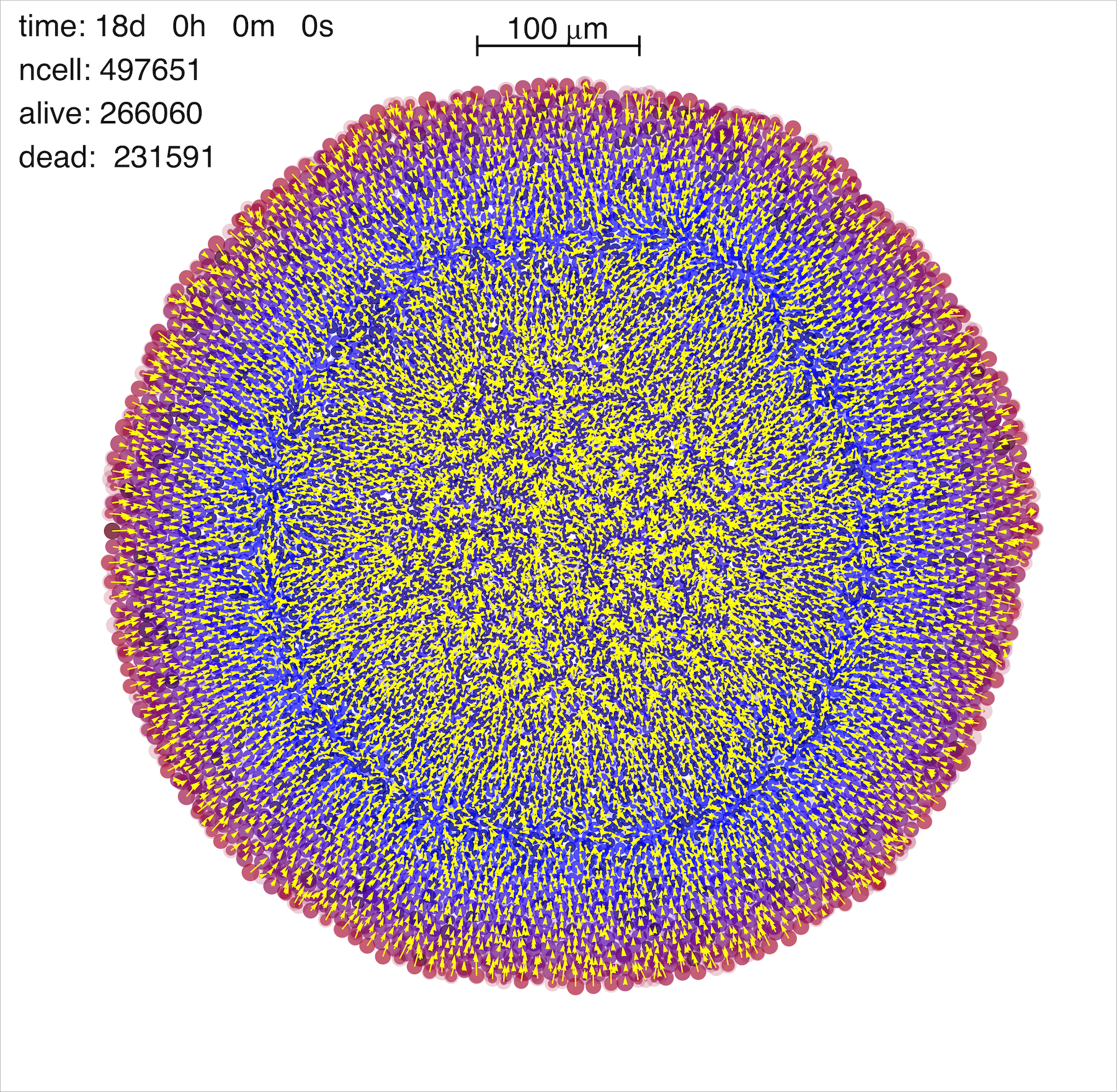

Supplement: Figure S17 — Extracellular glutamine concentration and flow at simulated time = 18 days. Cells are coloured to show the concentration of extracellular glutamine (color mapping, blue = low concentration, red = high concentration), while the yellow arrows show the flow of extracellular glutamine (arrow length proportional to flow intensity). (6.11 MB TIF) [file pone.0013942.s018.tif]

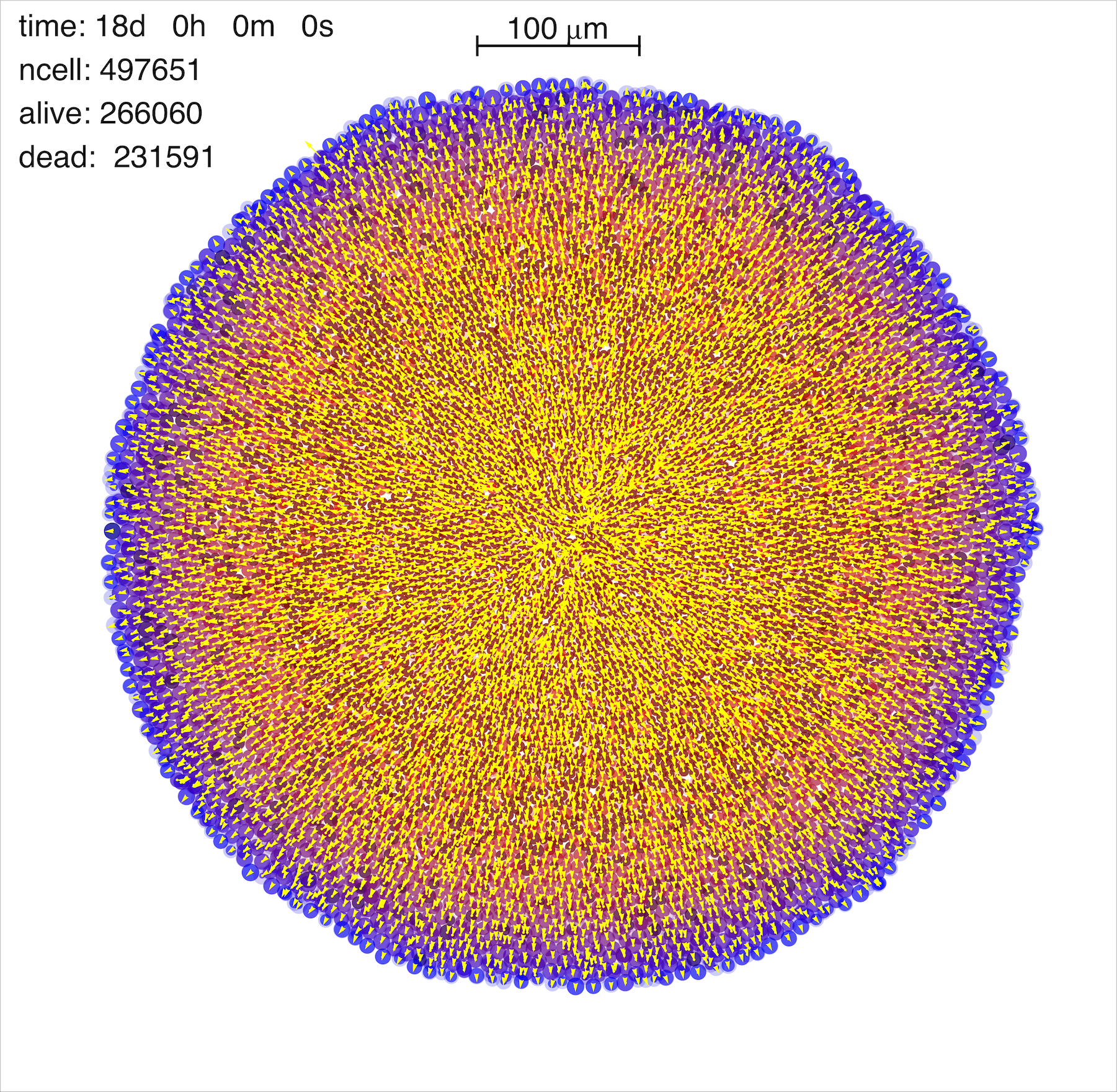

Supplement: Figure S18 — Extracellular lactate concentration and flow at simulated time = 18 days. Cells are coloured to show the concentration of extracellular lactate (color mapping, blue = low concentration, red = high concentration), while the yellow arrows show the flow of extracellular lactate (arrow length proportional to flow intensity). Lactate always flows outward in the simulation: this is a single snapshot taken after both glucose and glutamine have developed their split flow regime. (6.07 MB TIF) [file pone.0013942.s019.tif]

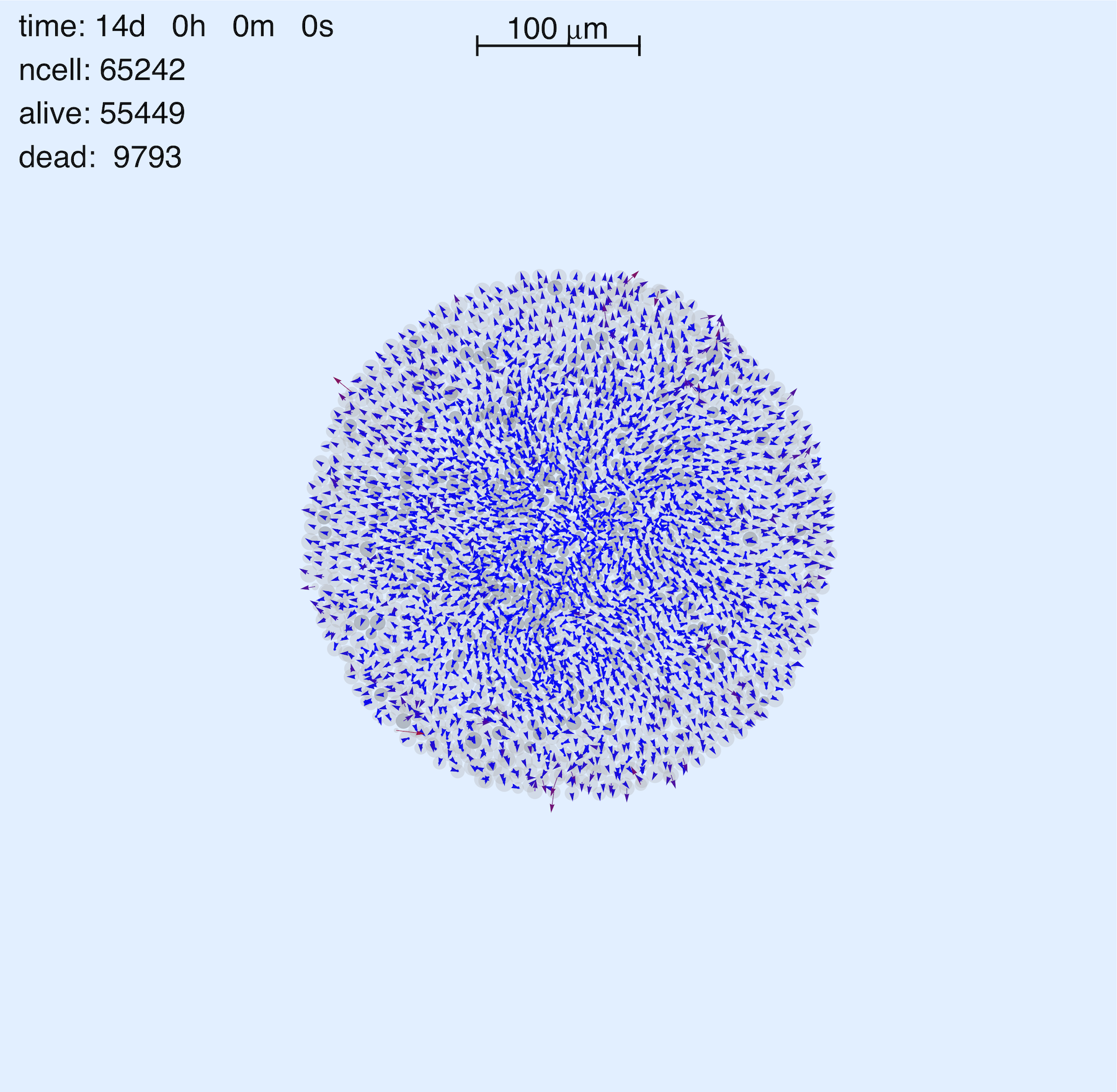

Supplement: Figure S19 — Velocity vectors projected on the plane of the slice at simulated time = 14 days. The vectors show the cells' motions in the plane of the slice (yellow arrows, arrow length proportional to flow intensity). Cells in the core perform complex looping motions, while cells in the viable rim always push outward. (1.62 MB TIF) [file pone.0013942.s020.tif]

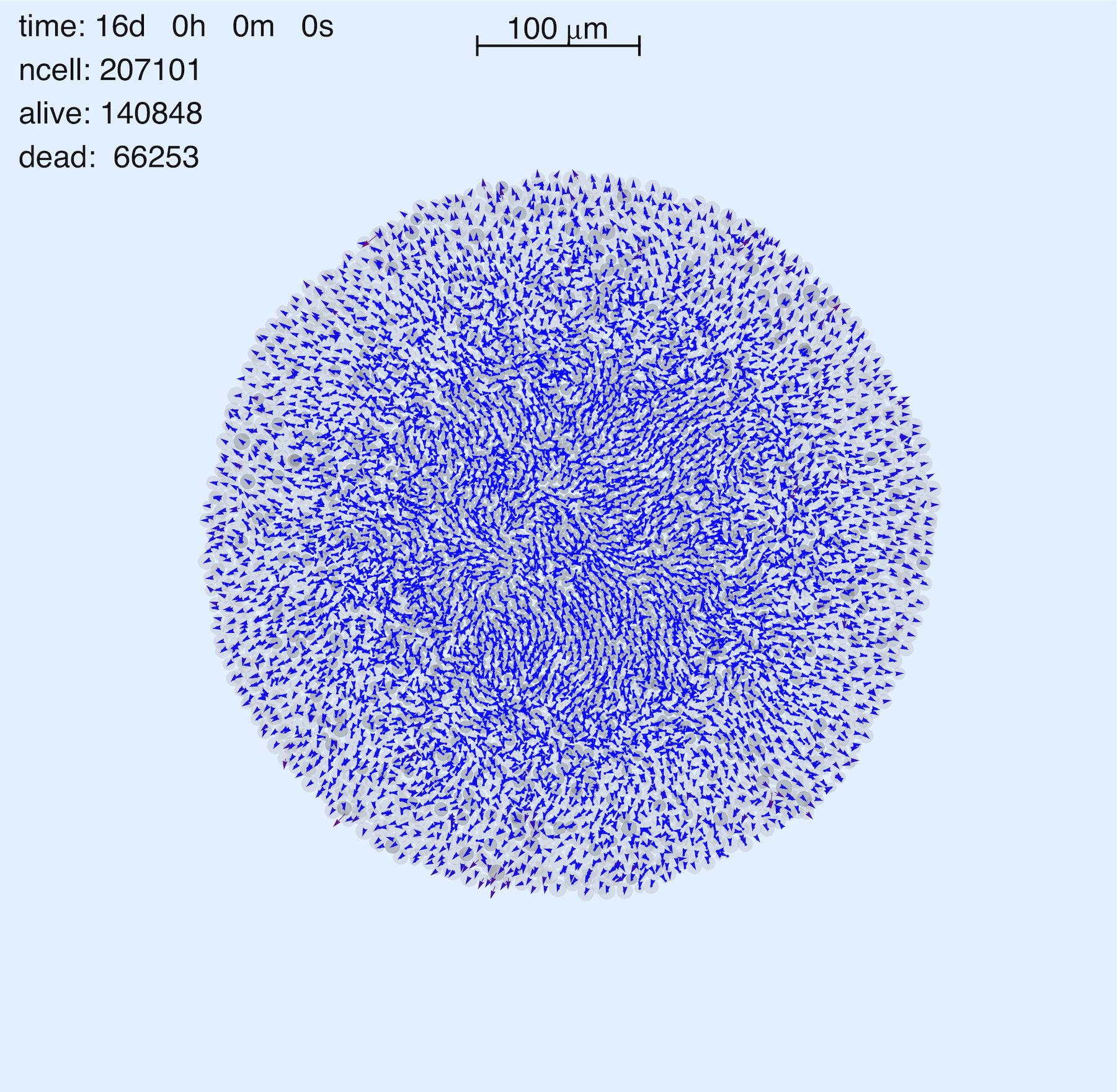

Supplement: Figure S20 — Velocity vectors projected on the plane of the slice at simulated time = 16 days. The vectors show the cells' motions in the plane of the slice (yellow arrows, arrow length proportional to flow intensity). Cells in the core perform complex looping motions, while cells in the viable rim always push outward. (3.08 MB TIF) [file pone.0013942.s021.tif]

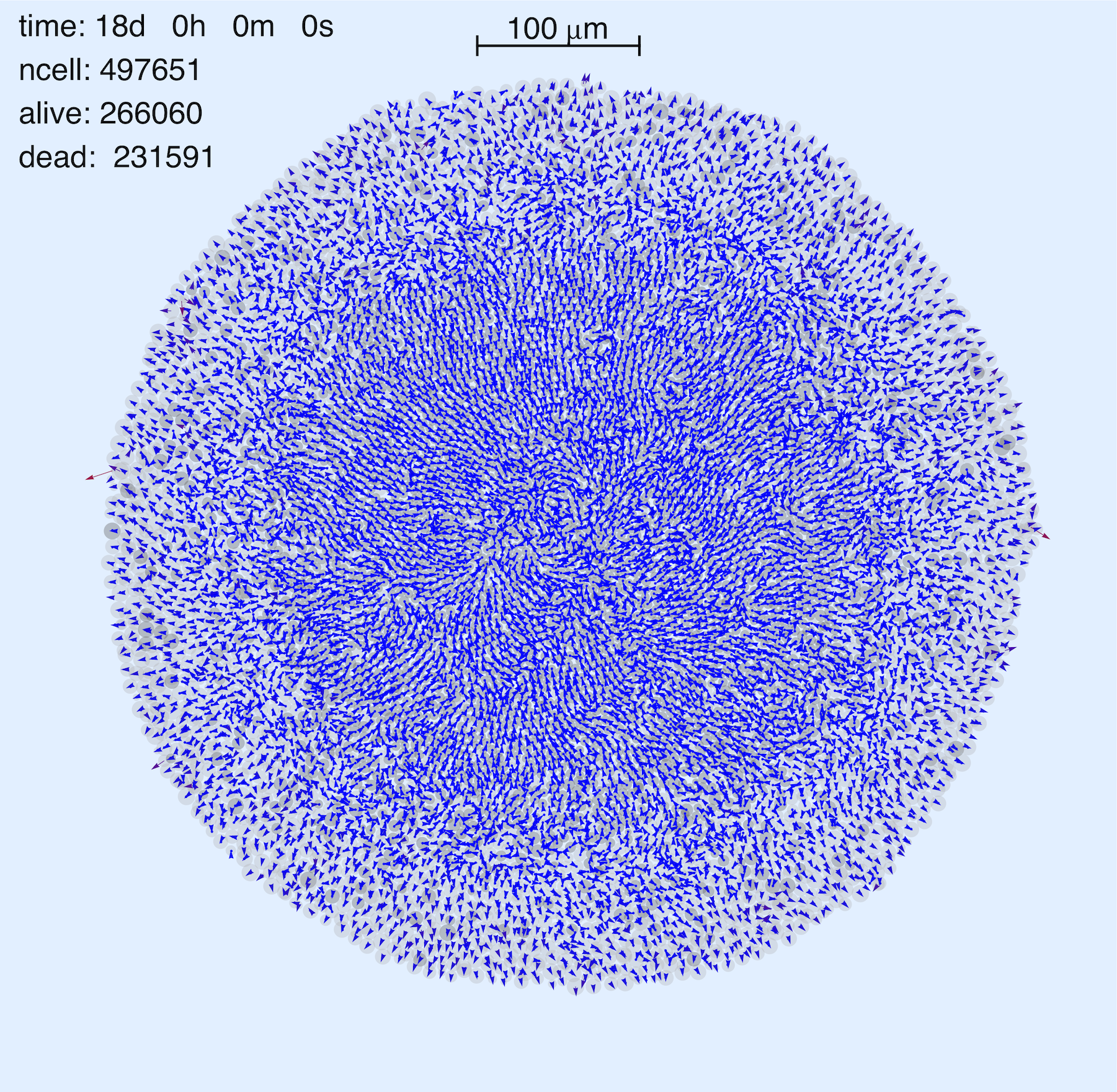

Supplement: Figure S21 — Velocity vectors projected on the plane of the slice at simulated time = 18 days. The vectors show the cells' motions in the plane of the slice (yellow arrows, arrow length proportional to flow intensity). Cells in the core perform complex looping motions, while cells in the viable rim always push outward. (5.00 MB TIF) [file pone.0013942.s022.tif]
